# Looking at the Camp: Paleolithic Depiction of a Hunter-Gatherer Campsite

**DOI:** 10.1371/journal.pone.0143002

**Published:** 2015-12-02

**Authors:** Marcos García-Diez, Manuel Vaquero

**Affiliations:** 1 Departamento de Geografía, Prehistoria y Arqueología, Universidad del País Vasco, Vitoria, Spain; 2 Departament de Història i Història de l’Art, Universitat Rovira i Virgili, Tarragona, Spain; 3 Institut Català de Paleoecologia Humana i Evolució Social (IPHES), Tarragona, Spain; Institute of Botany, CHINA

## Abstract

Landscapes and features of the everyday world were scarcely represented in Paleolithic art, especially those features associated with the human landscape (huts and campsites). On the contrary, other figurative motifs (especially animals) and signs, traditionally linked to the magic or religious conceptions of these hunter-gatherer societies, are the predominant themes of Upper Paleolithic art. This paper seeks to present an engraved schist slab recently found in the Molí del Salt site (North-eastern Iberia) and dated at the end of the Upper Paleolithic, ca. 13,800 years ago. This slab displays seven semicircular motifs that may be interpreted as the representation of dome-shaped huts. The analysis of individual motifs and the composition, as well as the ethnographic and archeological contextualization, suggests that this engraving is a naturalistic depiction of a hunter-gatherer campsite. Campsites can be considered the first human landscape, the first area of land whose visible features were entirely constructed by humans. Given the social meaning of campsites in hunter-gatherer life-styles, this engraving may be considered one of the first representations of the domestic and social space of a human group.

## Introduction

The iconography of Paleolithic art is largely made up of figurative depictions of animals [1, 2] and, less commonly, human figures. There is also a wide repertoire of non-figurative signs [3]. It is generally assumed that this imagery shows the importance of the animal world in the economic, social, and ideological systems of prehistoric hunter-gatherers [4]. Moreover, these animal figures exhibit the capacity to represent reality in a naturalistic style. The signs are commonly interpreted as symbolic representations with a heavy ideological burden. However, other interpretations offer a vision of Paleolithic art as social images linked to the realm of the everyday world [5, 6], challenging its association with a socially restricted religious sphere.

It seems that Paleolithic humans were less interested in representing features of the landscape. In particular, natural landscape features would be rarely represented and uncertain [7–9], let alone those forming part of the human landscape (huts and campsites). The few representations interpreted as huts [10–13] are formally undefined and open to alternative interpretations. The rarity of human landscape features in Paleolithic art is particularly striking if we bear in mind the importance of campsites in forager lifestyles, since they are the physical expression of hunter-gatherer social organization. Camps are made up of different social units that create their own household areas, which consist of one or more hearths and a dwelling structure [14–19]. In most cases, these dwelling structures are huts made of perishable materials, such as grass and branches, and can be constructed in a few hours. Most importantly, campsites are social spaces in which many interpersonal and socializing activities take place, including food-sharing and face-to-face interactions around hearths. As a spatial expression of a social group, campsites are particularly important in the study of human evolution because they may indicate the emergence of the communication skills and social structures typical of modern human behavior. In addition, campsites can be considered the first human landscape, the first area of land whose visible features were entirely constructed by humans.

Camps and huts were probably the first stage in the construction of the human landscape, but they are particularly elusive in archeological research. Although many sites have been interpreted as residential camps, the documenting of dwellings is less common. However, some remnants of dwelling structures have been identified from sharply defined archeological clusters, sometimes delimited by stone lines or post-holes [20–23]. These presumed dwellings tend to exhibit circular or oval plans. The characteristics of their walls and roofs are more difficult to infer, although the use of vegetable materials is often assumed. At Ohalo II, the plant remains found inside the structure are consistent with this interpretation [22]. Some exceptions are the mammoth bone dwellings from open-air sites in Eastern Europe [24]. Moreover, even when dwellings can be inferred, the identification of campsites encounters an additional problem: archeological sites are normally palimpsests in which it is difficult to establish whether the different structures were contemporaneous and therefore part of the same campsite. Refitting may contribute to solve this problem [25, 26], but the practice of recycling by Paleolithic groups highlights that refitting is currently a less straightforward evidence of synchronicity than was often assumed.

Although the signs in Paleolithic art have traditionally been regarded as devoid of any materiality, there are proposals arguing that some of them should be interpreted as natural landscape features (mountains, rivers, plants, etc). One of the most conspicuous examples is an engraved block from Abauntz cave (Spain), which displays what seems to be the landscape surrounding the cave [7]. Other alleged examples would be the marshlands represented in the El Pendo, Llonín and Gargas caves [8, 9].

Neither camps nor huts are usual in Paleolithic art, beyond the old interpretation of tectiforms as huts or roofs [27, 28]. This low representation of human and natural landscapes is in part due to the formal simplicity of these motifs (linear or geometric signs) and the absence of theoretical and ethnographic references to interpret these apparent non-representational depictions. Most of the purported depictions of residential architectures are found in Upper Paleolithic portable art from Central and Eastern Europe [10]. Some engravings have been interpreted as maps, hunting plans, or landscape features and certain arched motifs can recall shelters or huts [11]. The most commonly cited reference is an engraved ivory plaque from Mezhirich (Ukraine) [24, 12, 13]. This plaque is engraved with a series of motifs (zigzags, ladders, double lines, geometric forms) ordered along horizontal containing lines. In the central sector are four complex forms separated by rectangular motifs, which were interpreted as huts by Pidoplichko [13]. However, Marshack [12] challenged this interpretation and suggested that the two arched motifs forming the four purported dwellings looked like twin peaks with a possible astronomical body between them.

Our goal in this study is to present an engraving on a schist slab from a Magdalenian layer of the Molí del Salt site, which shows a series of dome-shaped motifs that can be interpreted as huts. We suggest that this engraving may be regarded as a naturalistic depiction of a hunter-gatherer campsite. This interpretation will be discussed using different kinds of ethnographic data.

## Materials and Methods

**Fig 1:**
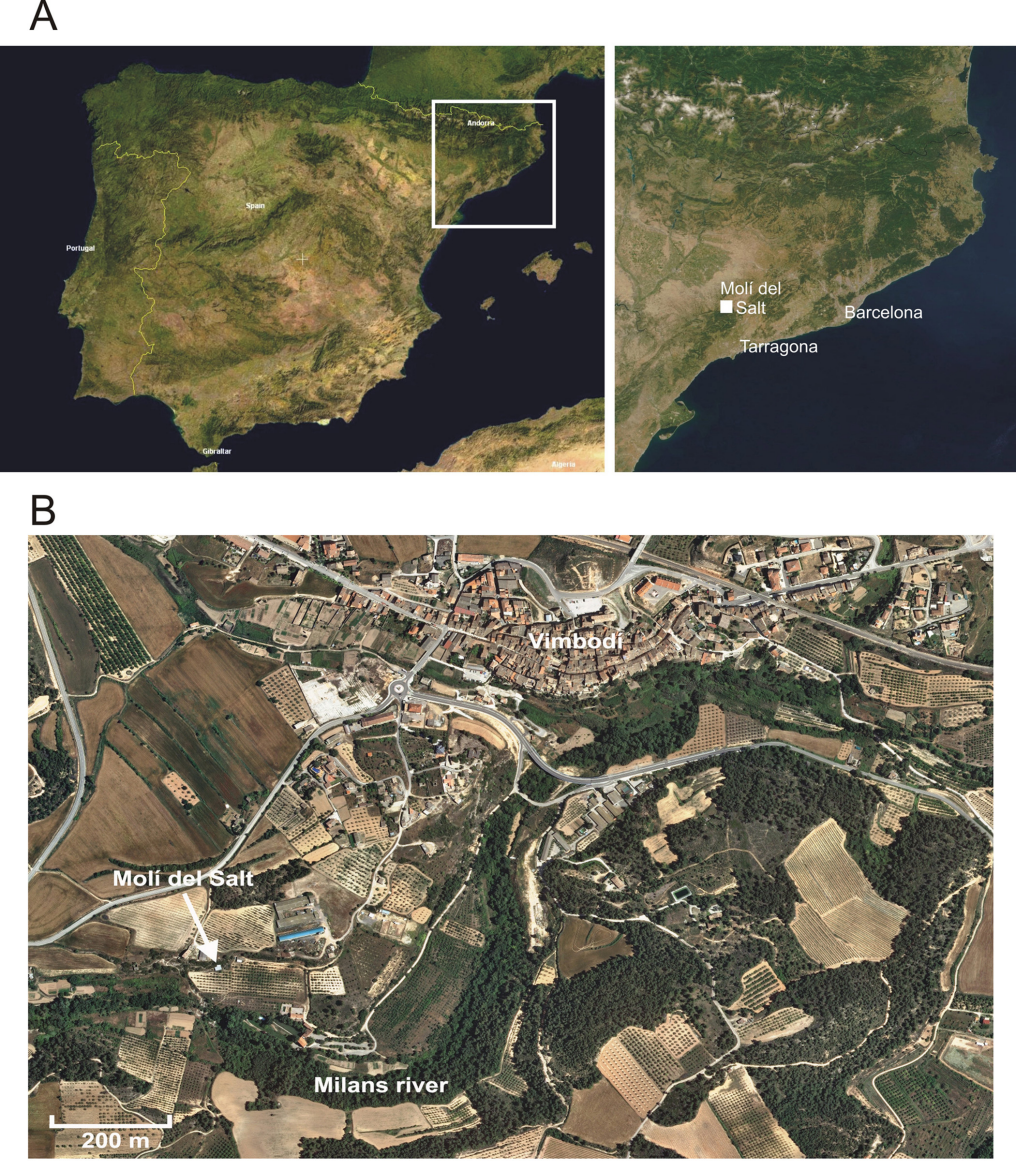
Molí del Salt location. Location of Molí del Salt in Northeastern Iberian Peninsula. A. NASA Satellite pictures. Licensed under Public Domain via Wikimedia Commons. B. Cartographic base (orthophoto) from the Institut Cartogràfic i Geològic de Catalunya (www.icgc.cat).

**Fig 2:**
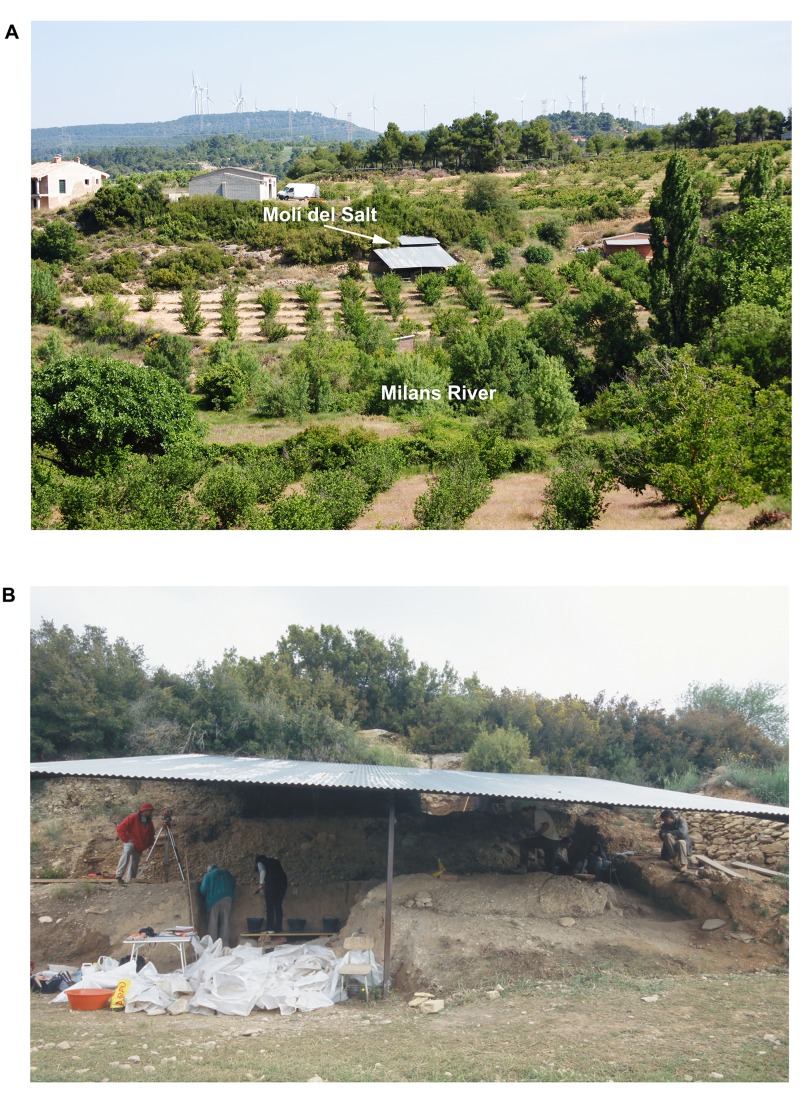
The Molí del Salt site. A. Image of the site from the opposite side of the Milans River. B. General view of the area that is currently being excavated.

The Molí del Salt (MS) is a rockshelter site in Northeastern Iberia (Vimbodí i Poblet, Spain) (Figs 1 and 2), 50 km west of the city of Barcelona, at 490 m above sea level on the left bank of the Milans, a small tributary of the Francolí river. The first excavations were carried out in 1999 and consisted of a test pit of 3 m^2^ that allowed documenting of the whole stratigraphic sequence. After the positive results yielded by the exploratory works, a research project was undertaken in 2001 and is still in progress. The stratigraphic sequence is 2.5 m thick and contains Mesolithic (level Sup) and Late Upper Paleolithic (Late Magdalenian) layers (units A and B) (Fig A in S1 File). Four main stratigraphic units have been identified, from top to bottom [29]:

–Superficial level (Sup). This unit is composed of dark gray sands, is poorly stratified, and is attached to conglomerate blocks from the previous unit. It has a variable thickness that increases toward the distal part of the deposit, where it reaches 20 cm. Mass wasting slope processes would be the dominant agent in the formation of this unit.
–Below level Sup, where a collapse episode is registered in which large conglomerate boulders fell and were subsequently incorporated into sand and red silt deposits. These deposits appear mainly in the sector closer to the wall of the rockshelter.
–Unit A. This unit is composed of approximately 70 cm of poorly stratified silty and sandy layers. Unit A includes three archaeological horizons (from top to bottom, Asup, A and A1). The prevailing sedimentary process is the mechanical weathering of sandstone from the rockshelter walls and ceiling.
–Unit B. This unit is 75-cm-thick composite of gravels and brown and dark yellow sand layers and is directly superimposed over the lutites of the substrate. It is a succession of lenticular beds subdivided into two horizons (B1 and B2). The sedimentary processes could be related to diffuse surface runoff water.

The Late Upper Paleolithic units have been dated between ca. 13 and 15 kyr cal BP [30] (S1 File). Both the faunal and lithic assemblages exhibit the characteristics typical of the Late Upper Paleolithic sites in Mediterranean Iberia. Rabbit is the most represented species at every archeological level, although some macromammal remains (ibex, red deer, wild boar, lynx, fox, and badger) have also been documented. Flint is the dominant raw material in lithic assemblages and the toolkits are composed of the artifacts commonly found in Late Magdalenian sites: endscrapers, backed artifacts, truncations, denticulates, burins, and borers. Thirteen portable art objects with engravings have so far been recovered. Nine of them are schist slabs, but representations have also been identified on three limestone cobbles and one bone fragment. Most of these objects were found in unit A and show animal figures and schematic motifs with the typical late-Paleolithic stylistic patterns [31]. The art object presented in this paper is an engraved schist slab found at the top of unit B (level B1) during the 2013 field season. A bone fragment located 82 cm from the engraved slab in the same stratigraphical horizon has been dated to 11,880±50 BP (14,062–13,498 cal BP at 95.4% probability).

The engraved slab (ref. num. MS13 B1 E35/22) is housed in the Institut de Paleoecologia Humana i Evolució Social (IPHES), Marcel·lí Domingo s/n, Campus Sescelades URV, 43007 Tarragona, Spain. Excavation permit for the 2013 field season was issued by the General Director of Archives, Libraries, Museums, and Heritage of the Generalitat de Catalunya (03/21/2013, Exp. Num. 437 K121 N935-2013/9524). The landowners gave also their permission to carry out the work. For the study, a direct record of the engraved surfaces was performed using a microscope [32]. A stereoscopic microscope (Nikon SMZ800) camera for capturing images (Nikon DS-Fi2), and image processing software (NIS-Elements F4.00.06) was used for the technological study. The analysis of the technical parameters was based on the work of d'Errico [33] and Fritz [34]. In general, the object is macroscopically well preserved, but some areas of the surfaces and the inside of the grooves are covered by deposits of concretion, limiting the analysis (especially in graphic units C and D of the upper face).

## Results

**Fig 3:**
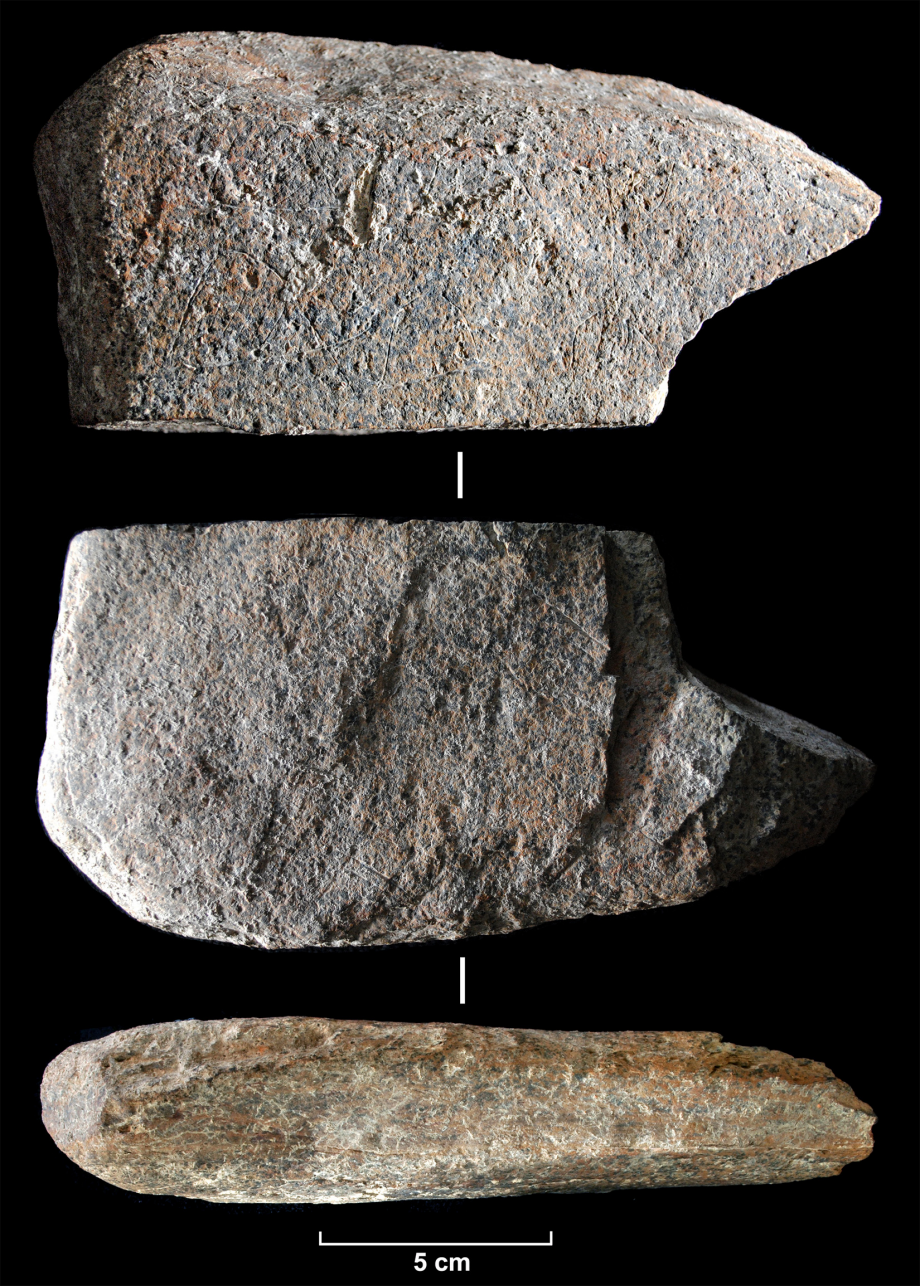
The engraved slab. Photograph of the engraved schist slab from Molí del Salt.

**Fig 4:**
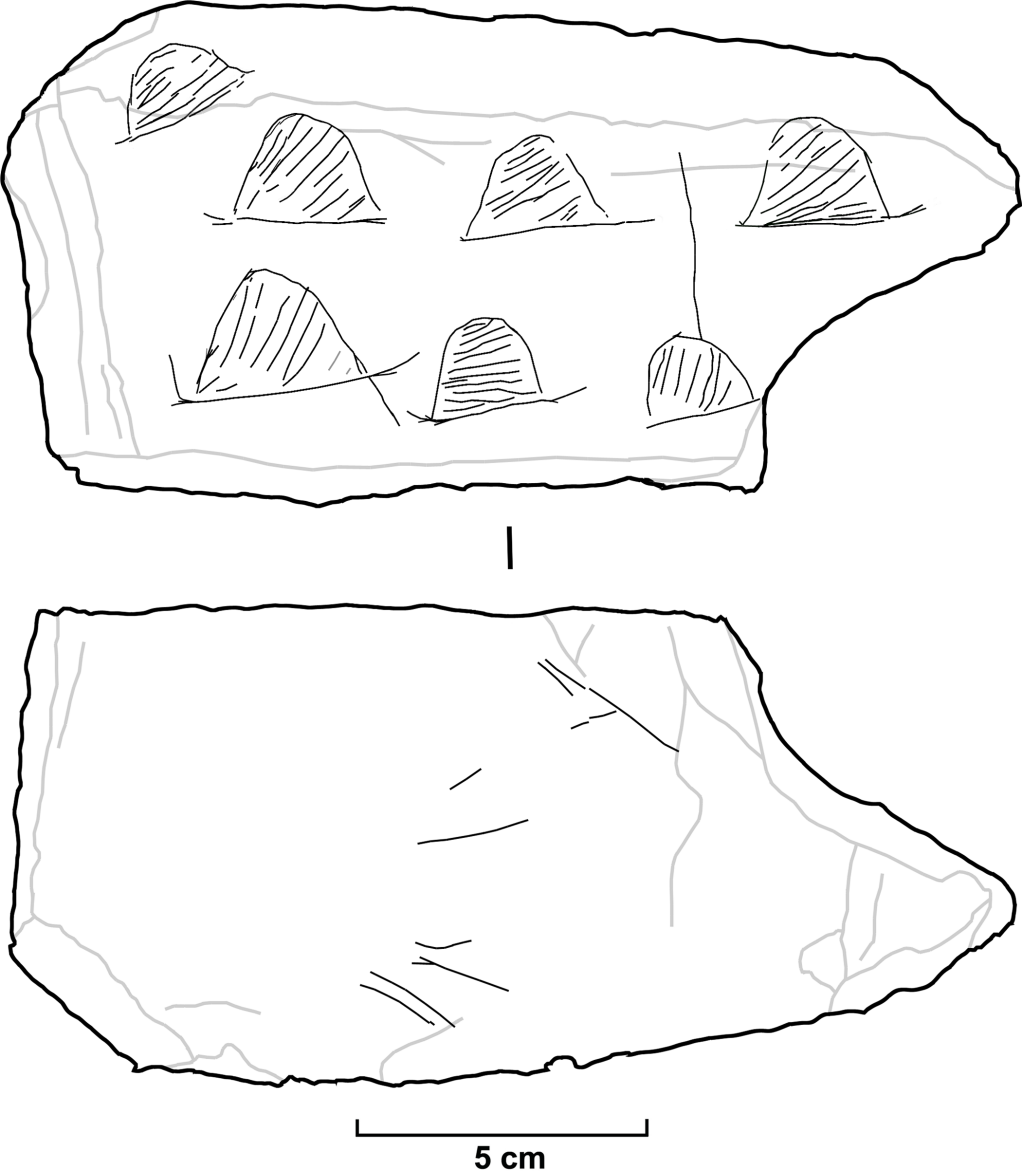
Drawing of the engraved slab.

**Fig 5:**
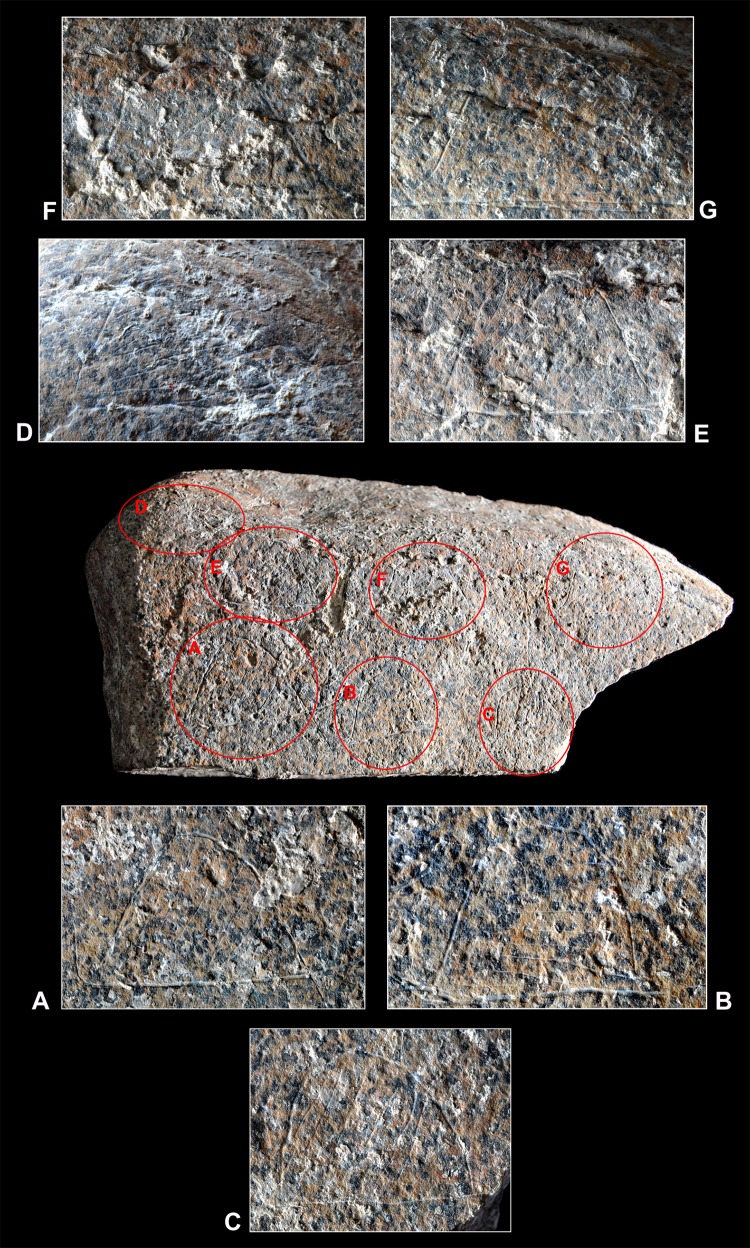
Close-ups of the motifs. Photograph of the engraved side with close-ups of the seven semicircular motifs. No graphic scale.

The slab shows a trapezoidal morphology, and its maximum dimensions are 18 cm wide, 8.5 cm high, and 3.6 cm thick (Figs 3, 4 and 5). Grauvaquic schist is from the Paleozoic formations 4 km south of the site and schist slabs are abundant in the alluvial deposits in front of the rockshelter. There are seven graphic units in the upper surface, while only a small set of lines are recognized in the lower one. The graphic units correspond to seven semicircular motifs whose interior was filled by straight parallel lines (individualized descriptions and images of each unit can be found in S2 File). The geometric structures are constructed from two different contour lines–one straight and one curved–that define the convex character. The straight line defines the lower part of the motif, so all structures have the same disposition. The interconnection between the two structural lines is blurred, as neither contour line touches or exceeds the other (normally, the bottom line exceeds the ends of the curved line). The number of internal lines varies between 7 and 11 (Table 1), mostly covering the entire interior space. The internal lines, in general, do not reach the contour lines, and they show a horizontal (two cases) or oblique (five cases, three of which show a marked tendency to vertical) disposition. The size of the graphic units varies between 43 and 20 mm in width and between 22 and 14 mm in height. If we consider only the semicircular shape, without considering the appendices of the lower contour line, the dimensions range between 30 and 18 mm in width and 22 and 14 mm in height.

**Table 1:** Morphological, metric and technical attributes of the seven semicircular motifs identified in the Molí del Salt slab.

Figure	External morphology	Internal lines		Size of the semicircular motif		Technique	Contour	Characterization of grooves		Corrections
		Number	Arrangement	Width (mm)	Height (mm)			Morphology	Depth	
A	Semicircular	8	Transverse	30	22	Incised engraving	Simple	U	Superficial and medium	Yes
B	Semicircular	12	Horizontal	20	18	Incised engraving	Simple	U	Shallow and medium	Yes
C	Semicircular	7	Vertical	18	15	Incised engraving	Simple	U	Shallow and medium	Yes
D	Semicircular	8	Horizontal	22	14	Incised engraving	Simple	U	Superficial and medium	
E	Semicircular	10	Transverse	26	19	Incised engraving	Simple	U	Shallow and medium	Yes
F	Semicircular	11	Transverse	25	18	Incised engraving	Simple	U	Shallow and medium	Yes
G	Semicircular	11	Transverse	26	19	Incised engraving	Simple	U	Shallow and medium	Yes

The analysis of the semicircular motifs shows various aspects related to the creative process, although we must take some caution due to taphonomic conditions that prevent a complete reading of the lines. Curved contour lines were executed with several strokes (between at least two and seven), and three parts can be distinguished: a) the left side, made from the bottom up (three cases), from the top down (two cases), and bidirectionally (one case); b) the distal sector, carried out from right to left (two cases), left to right (one case), and bidirectionally (one case); and c) the right side, made from top to bottom (five cases). The rectilinear contour line was drawn with one stroke, although small strokes are added in most ends to define its length or shape, forming diffuse endings. There is no clear-cut interconnection between the two structural contour lines, as the bottom line usually exceeds, to varying degrees, the limit defined by the curve. The direction of the interior lines tends to show a variable pattern: two opposite directions (up-down or left-right) and bidirectional patterns combined in one case, bidirectional and one direction in two cases, two directions in two cases, and unidirectional patterns in two cases. Overlapping lines indicate that the curved contour line was made from left to right; the lower straight line was usually drawn after the curved contour line; and the interior lines were made after the contour lines. The grooves show a U-section, have a superficial or moderate depth (especially at the ends of the strokes that make up the contour lines and inner lines) and a variable width between 0.4 and 1 mm.

The semicircular motifs are arranged in three levels. The lower and middle levels present three motifs arranged according to the same baseline. The motifs of these two levels tend to be aligned (especially those on the left side) or turned to the right (especially those on the right side). The lower level shows a progressive reduction in the size of the motifs from left to right (from 30 to 18 mm in width and from 22 to 15 mm in height). The third level is composed of a single motif on a downward surface and is turned to the left. The left side of the slab shows a decrease in motif size from bottom to top because the motif from the third level is narrower and shorter than the first motifs from the other two levels. The distribution, organization and size of these motifs suggest that this is an integrated composition constructed for generating a progressive visual sensation of depth (both in its vertical–from bottom to top–and horizontal–from left to right–reading) based on gradients. The oblique disposition in the relationship between the motifs of the lower and middle levels increases this sensation.

The thematic monotony, the trends in the pattern of engraving (mainly the execution order between the lines and the composition of lines), the diversity in the direction of the engraving conditioned by the possibility of changing the position of the support, the technical homogeneity, and the organization and distribution of the graphic units allow us to argue that the motifs form a compositional unity engraved in a short timeframe.

## Discussion

We hypothesize that the seven semicircular motifs in the MS engraving represent dwellings or huts. In addition, the close formal, metric, and technical linkages among these motifs, as well as their distribution in the graphic field, indicate their compositional association and their execution in a short time. To support the interpretation of the MS engraving as a campsite, we will focus on three aspects for which we have ethnographic information: the outline of the huts, their proportions, and the number of huts in a campsite. The use of ethnographic information in archeological interpretation has been common since the 1970s. This is based on the assumption that there are some analogies between present and past societies that produce similar archeological outcomes. Hunter-gatherer architecture is strongly conditioned by one of the characteristics associated to most hunter-gatherer societies: residential mobility. According to this assumption, mobile hunter-gatherers will show common traits in their architectural patterns, regardless their historical contexts.

### Hut morphology

Regarding the morphology, ethnographic data indicate that mobile peoples tend to build domed circular or semicircular houses [35–38] (S1 Fig). These huts are normally made using a frame of wooden poles covered with some sort of roofing material (grass, brush, hides). Dome-shaped dwellings–also known as beehive huts and wigwam or wickiup–are more common among hunter-gatherers [11, 12, 14, 15] than the conical tepees sometimes used as a reference for the reconstruction of Paleolithic dwellings [25]. Domes are self-supporting structures and do not need inner supportive elements, providing an entirely free interior space. They are more stable and resistant to physical forces and enclose the largest volume with the smallest structure [36, 39]. Although they are difficult to subdivide into compartments and cannot be enlarged without removing structural elements [36], these drawbacks seem of little concern for highly mobile people.

The construction of circular dome-shaped structures may be completed in a short time and they are therefore particularly appropriate in short-term camps. Sometimes, the use of dome-shaped dwellings exhibits a seasonal pattern; they are preferred in the most mobile phase of the settlement system, while other lodges are built in the most sedentary phase. As societies become sedentary and their sociopolitical organization becomes more complex, rectilinear houses tend to be more common [35–38, 40, 41]. It should be stressed that some societies that have adopted rectilinear lodges still maintain dome-shaped structures for the building of particularly ephemeral huts, such as sweatlodges and menstrual huts (see examples at [42, 43]). It seems, therefore, that temporary dome-shaped dwellings are closely linked to the mobility and social structure typical of hunter-gatherers. Dousset [44] notes how the Ngaatjatjarra quickly abandoned the Western-style rectangular houses provided by the Australian administration and returned to the dome-shaped traditional *wiltjas*, even recycling the construction materials from the rectilinear lodges. According to Dousset, such allegiance to the traditional dwellings is related to the preservation of the social relations characteristic of the hunter-gatherer life-ways.

### Hut proportions

**Fig 6:**
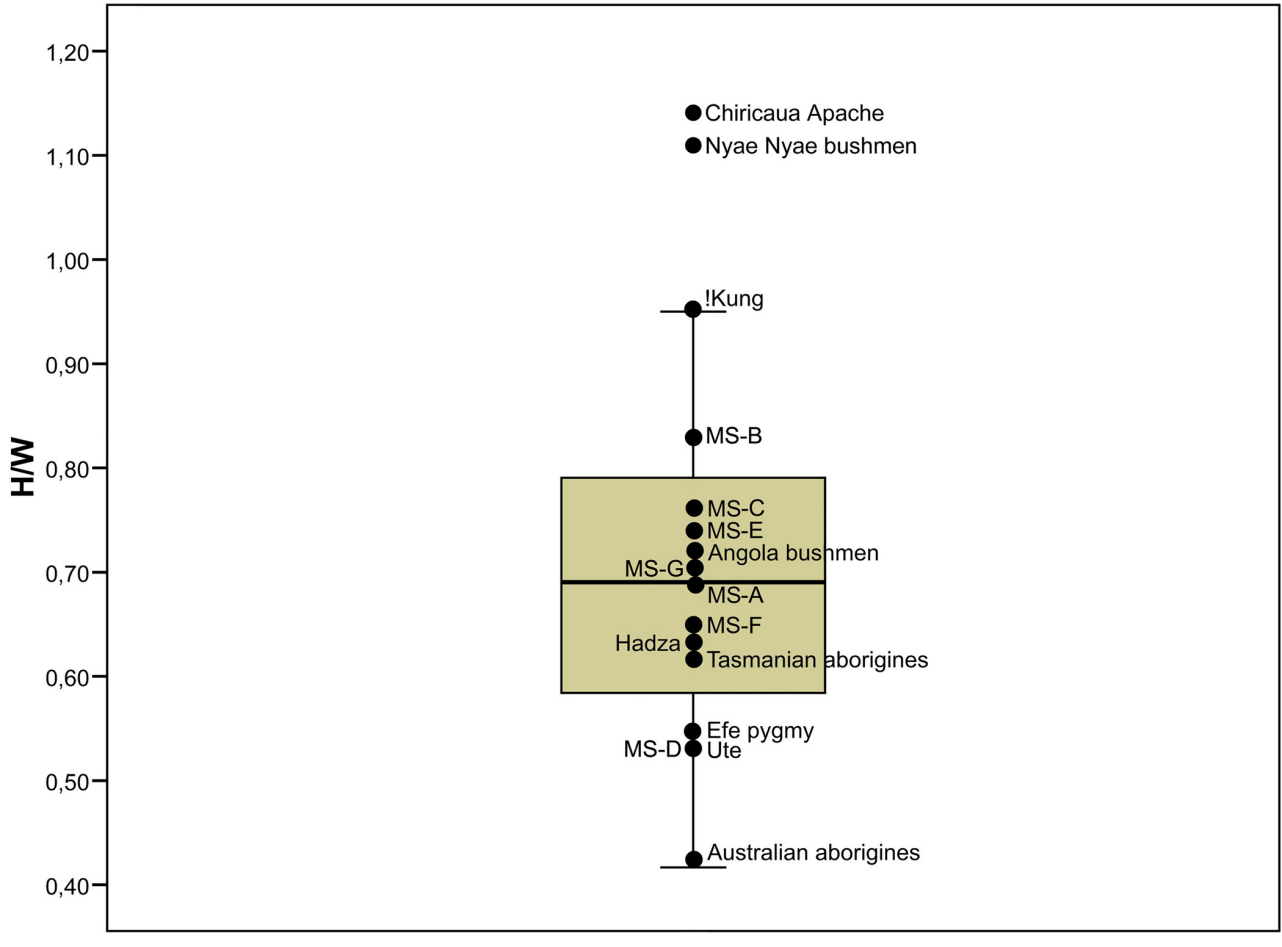
Slenderness ratio of ethnographic and MS huts. Box and dot plot of slenderness ratio of ethnographic huts and engraved huts (MS) from the Molí del Salt slab.

Building techniques change according to climate and the available materials. Intra-group variability also depends on factors such as the anticipated occupation length, the composition of the household unit, and the seasonal weather variability. The height/width ratio (the slenderness ratio) basically defines shape variability. This is a measure commonly used in architecture and engineering [45, 46]. Although the information on hut size and proportions is scanty in the ethnographic literature, some data suggest that these domed dwellings tend to exhibit similar proportions. In general, they are slightly wider than they are high or as high as they are wide. According to Opler [47], the wigwams of the Chiricaua Apache are among the few cases of domes that are higher than wide: “…*eight feet high at the center and approximately seven feet in diameter*” (that is, approximately 243 x 213 cm). The domed houses of the Ute “…*were about eight feet high and 15 feet in diameter*…” (243 x 457 cm) [48]. The! Kung huts are “…*slightly under 2 m in height and about 2 m in diameter*” [15]. Marshall [49] reports similar proportions for the bushmen of the Nyae Nyae region: four to five feet wide and five feet high (122/152 x 152 cm). Quoting Vedder [50], Urquhart [51] notes that the huts of the Southwestern Angola bushmen are six to eight feet wide and five feet high (183/243 x 152 cm). The wet season huts of the Hadza are reported to be “…*2 to 3 m in diameter and about 1*.*6 m in height*” [14]. Mean values for the diameter and height of the Efe pygmy dwellings are, respectively, 254 and 138 cm, although Fisher and Strickland [11] indicate that the two dimensions exhibit considerable variation. Dome-shaped dwellings were larger among the Tasmanian aborigines, “*measuring some 4 m in diameter and 2*.*5 m high*” [52]. Dwellings of Australian aborigines also tend to be wider than they are high [53, 54]. According to Memmot [53], they were up to 3.6 m in diameter, whereas the heights were consistently 1.2 to 1.5 m. Moreover, beehive huts made by some nomadic pastoralists are similar to those of hunter-gatherers living in the same region: “*Some of the sleeping huts of the Dimba are very similar to those of the Bushmen*. *They are about seven feet in diameter and five feet tall…*” (213 x 152 cm) [51]. Although the functionally specific structures (sweatlodges, menstrual huts) tend to be smaller than the residential domes, they exhibit similar proportions, as shown, for example, by the sweatlodges of the Sahaptins, which are “…*approximately two meters in diameter and 1*.*2 meters high*” [55]. The slenderness ratio of these ethnographic dome-shaped dwellings ranges between 1.14 and 0.33. The slenderness ratio of the MS huts ranges between 0.53 and 0.82 (mean = 0.70 ± 0.09) and is fully consistent with the values recorded in ethnographic contexts (Fig 6).

### Number of huts

Band size displays a high degree of variability, both at the intra-group and inter-group levels [56, 57, 58]. Dynamics of fission and fusion related to the seasonal variability in resource distribution characterize most hunter-gatherer bands. In addition, there are other factors related to the primarily exploited resources. Band sizes of mobile peoples depending on terrestrial plants are smaller (12 ± 4 during the most dispersed phase of the settlement system and 34.1 ± 10.8 during the most aggregated) than those of mobile peoples primarily dependent upon hunting of terrestrial animals (16.3 ± 5.1 during the most dispersed phase and 46.7 ± 18.2 during the most aggregated phase) [56]. The number of dwelling structures in a hunter-gatherer camp is also highly variable. This number depends not only on the band size or the amount of familiar units forming the band but also on the socio-economical organization [56]. When taking into account the different hunter-gatherer systems distinguished by Binford (Table 2), the groups characterized as ‘generic hunter-gatherers’ show 2.6 ± 1.1 households per camp during the most dispersed phase of the settlement system and 7.6 ± 3.9 during the most aggregated phase. The seven huts in the MS engraving fit perfectly with this mean number of households in aggregation camps and are close to the mean values of other hunter-gatherer systems.

**Table 2:** Mean number of dwelling structures in hunter-gatherer campsites. Data from Binford [56].

System state classification	Smallest residential seasonal camps	Largest residential seasonal camps
Mounted hunters	5.5 ± 2.2 (n = 19)	24.9 ± 13.4 (n = 20)
Horticulturally augmented cases	2.6 ± 1.1 (n = 13)	9.5 ± 6.6 (n = 16)
Mutualists and forest products specialists	4.1 ± 2.1 (n = 21)	9.6 ± 5.5 (n = 21)
Generic hunter-gatherers	2.6 ± 1.1 (n = 72)	7.6 ± 3.9 (n = 77)
Generic hunter-gatherers with instituted leadership	2.04 ± 0.7 (n = 13)	5.6 ± 2.9 (n = 17)
Wealth-differentiated hunter-gatherers	2.4 ± 1.1 (n = 27)	9.8 ± 9.7 (n = 47)
Internally ranked hunter-gatherers	1.3 ± 0.4 (n = 4)	14.02 ± 15.1 (n = 19)

**Fig 7:**
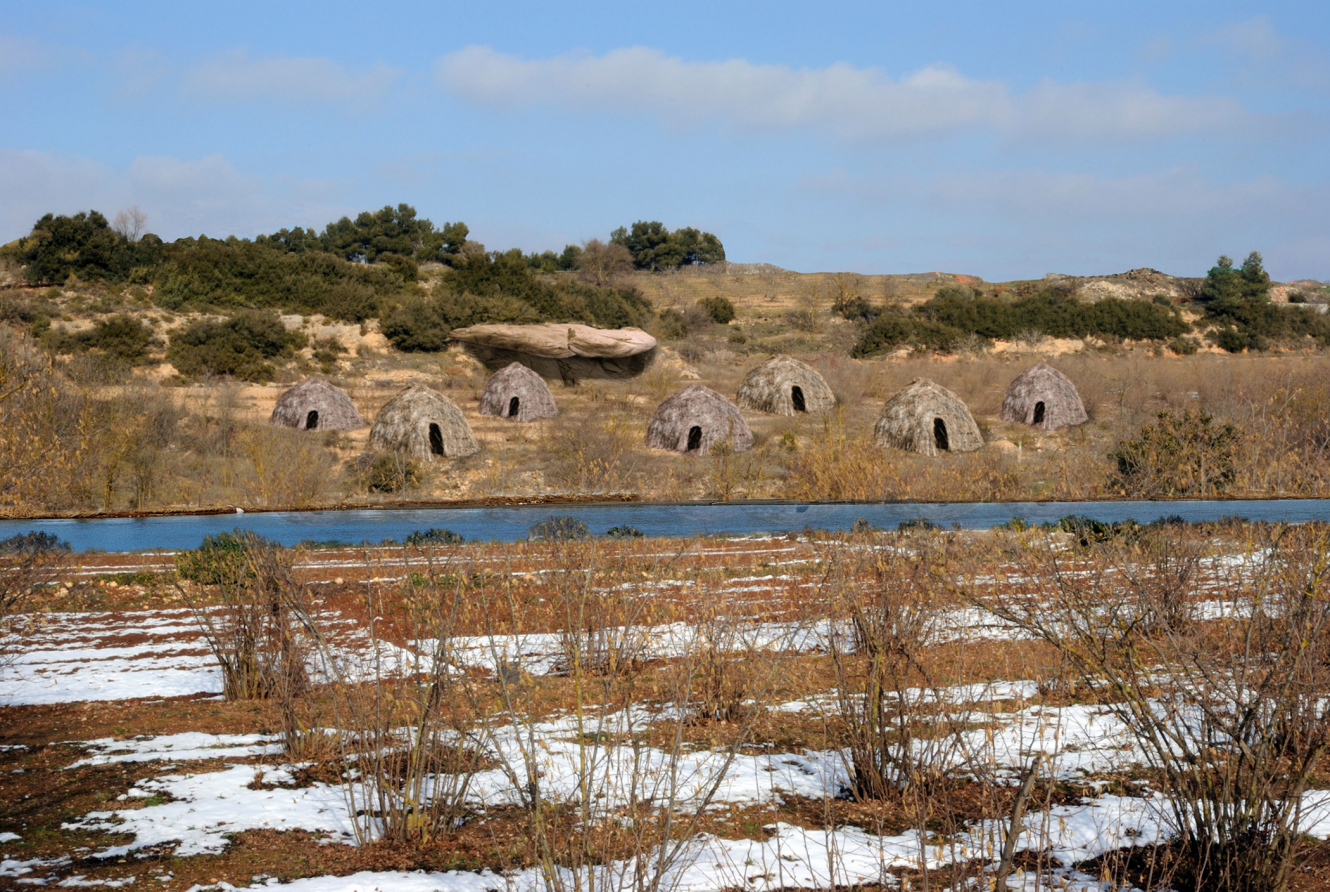
The Molí del Salt campsite. Digital reconstruction of the Molí del Salt campsite, by Luis Alberto Marcos. Hut design from Apache Wickiup, Edward Curtis, 1903 by Library of Congress. Licensed under public domain via Wikimedia Commons.

The archaeological context of the site reinforces this interpretation of the MS engraving. In front of the rockshelter, there is a plain descending smoothly to the river, which is located 100 m south of the site. This setting has been documented for some open-air Magdalenian sites interpreted as campsites [25, 59, 60] and is characterized by well-defined clusters of remains corresponding to domestic areas. In the plain in front of the MS rockshelter, surface archeological remains are abundant and thousands of artifacts have been recovered over the years. Most of them exhibit a fresh appearance, indicating that they were not displaced from the rockshelter but correspond to in situ archeological deposits below the current surface. Moreover, they exhibit the same technological and typological characteristics than the lithic assemblages from the rockshelter. This suggests that alongside the rockshelter, there was also an open-air settlement in the plain next to the river. It seems likely, therefore, that the engraving represents a reality that was in front of the artist’s eyes at the moment of the depiction (Fig 7).

The MS engraving shows the possibility of interpreting some of the signs of Paleolithic art as representations of tangible images from real perceptions. Ethnographic data support its interpretation as the realistic representation of architectonical structures. The convergences in the engraving process, as well as the distribution and association of the seven motifs allow us to interpret this composition as the representation of a hunter-gatherer campsite. Considered as a ‘frozen and photographic image’ of a human landscape, the MS evidence offers a different vision of Paleolithic art based on a social image of art linked to the realm of the everyday life. This engraving is one of the few examples of architecture and anthropic landscape art so far documented in Paleolithic archeology. Unlike other purported examples of landscape depictions, it mainly represents a human landscape, suggesting that the human world was the main concern of the artist. Given the social meaning of campsites in a hunter-gatherer organization, it can be considered one of the first artistic representations of the domestic and social space of a human group.

## Supporting Information

S1 Fig Ethnographic huts. Ethnographic examples of dome-shaped dwellings in hunter-gatherer campsites. **A**. Apache Wickiup, Edward Curtis, 1903 by Library of Congress. Licensed under Public domain via Wikimedia Commons. **B**. Bushmen San. Licensed under Public domain via Wikimedia Commons. **C**. Hut Eastern Arrernte by Herbert Basedow—National Museum of Australia. Licensed under Public domain via Wikimedia Commons. **D**. Apache Indian Kan or brush house, ca.1900 (CHS-3581) by Pierce, C.C. (Charles C.). Licensed under Public domain via Wikimedia Commons. **E**. Baldwin Spencer seated with the Arrernte elders, Alice Springs, Central Australia, 1896.—Google Art Project by Walter Baldwin Spencer and Francis J Gillen.
(PDF)

S1 File The Molí del Salt site. Site description, chronology and archeological context.
(PDF)

S2 File Technical analysis. Individualized description of the graphic units.
(PDF)
