# Supplementary material for: Looking at the Camp: Paleolithic Depiction of a Hunter-Gatherer Campsite
Source: PLoS One. 2015 Dec 2;10(12):e0143002. doi: 10.1371/journal.pone.0143002 (PMC4668041; doi:10.1371/journal.pone.0143002)
Supplement: S1 Fig — Ethnographic examples of dome-shaped dwellings in hunter-gatherer campsites. A. Apache Wickiup, Edward Curtis, 1903 by Library of Congress. Licensed under Public domain via Wikimedia Commons. B. Bushmen San. Licensed under Public domain via Wikimedia Commons. C. Hut Eastern Arrernte by Herbert Basedow—National Museum of Australia. Licensed under Public domain via Wikimedia Commons. D. Apache Indian Kan or brush house, ca.1900 (CHS-3581) by Pierce, C.C. (Charles C.). Licensed under Public domain via Wikimedia Commons. E. Baldwin Spencer seated with the Arrernte elders, Alice Springs, Central Australia, 1896.—Google Art Project by Walter Baldwin Spencer and Francis J Gillen. (PDF) [file pone.0143002.s001.pdf]

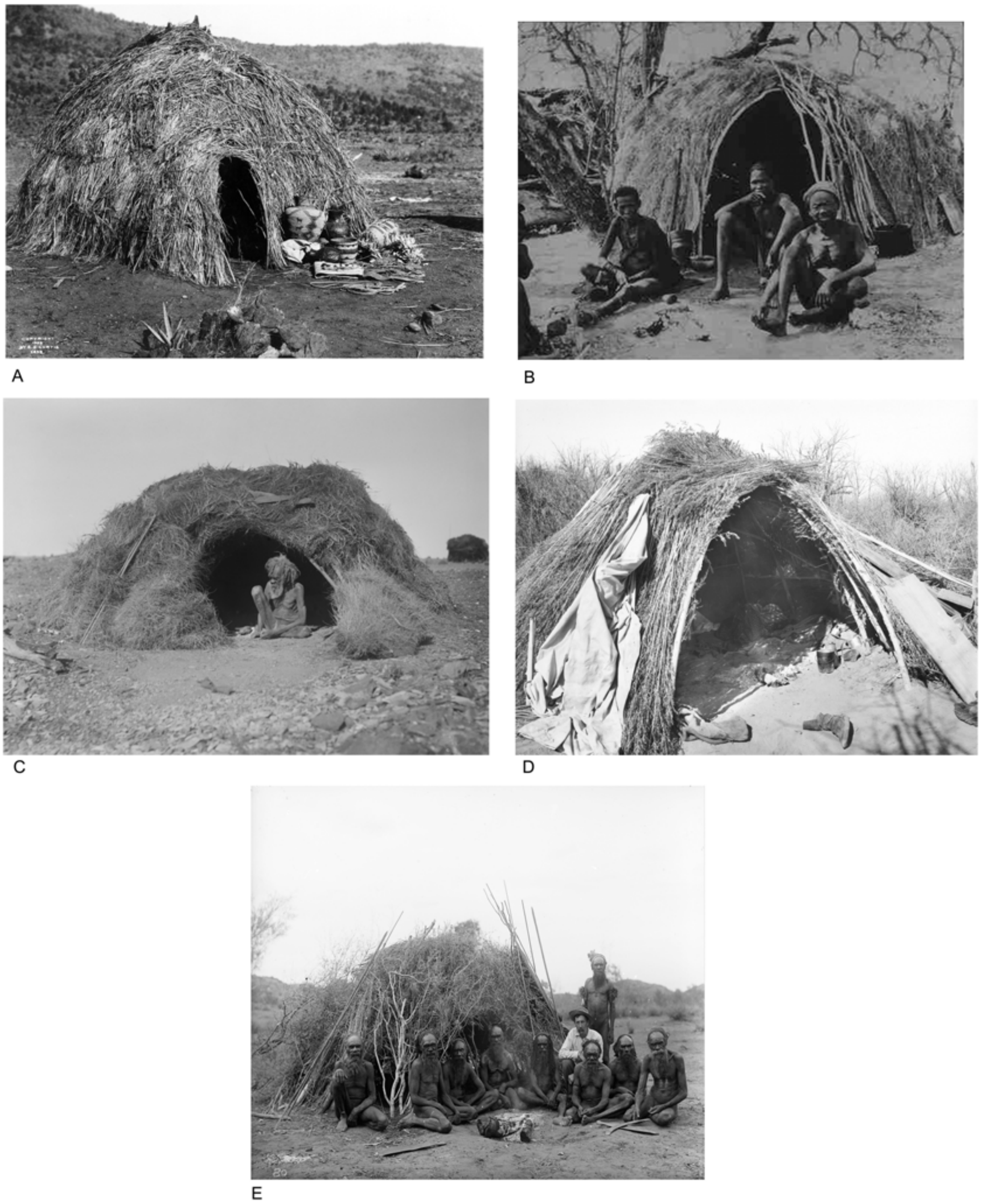

S1 Fig. Ethnographic examples of dome-shaped dwellings in hunter-gatherer campsites. **A**. Apache Wickiup, Edward Curtis, 1903 by Library of Congress. Licensed under Public domain via Wikimedia Commons. **B**. Bushmen San. Licensed under Public domain via Wikimedia Commons. **C**. Hut Eastern Arrernte by Herbert Basedow - National Museum of Australia. Licensed under Public domain via Wikimedia Commons. **D**. Apache Indian Kan or brush house, ca.1900 (CHS-3581) by Pierce, C.C. (Charles C.). Licensed under Public domain via Wikimedia Commons. **E**. Baldwin Spencer seated with the Arrernte elders, Alice Springs, Central Australia, 1896. - Google Art Project by Walter Baldwin Spencer and Francis J Gillen.
