# Supplementary material for: Looking at the Camp: Paleolithic Depiction of a Hunter-Gatherer Campsite
Source: PLoS One. 2015 Dec 2;10(12):e0143002. doi: 10.1371/journal.pone.0143002 (PMC4668041; doi:10.1371/journal.pone.0143002)
Supplement: S1 File — Site description, chronology and archeological context. (PDF) [file pone.0143002.s002.pdf]

S1 File. Site description, chronology and archeological context

The Molí del Salt site is located in the village of Vimbodí i Poblet, 50 km west of the city of Barcelona (Northeastern Iberia), at 490 m above sea level on the left bank of the Milans, a small tributary of the Francolí river. Its UTM coordinates are X=336532.5 Y=458446.5 (ETRS89 system). It is a south-facing rockshelter in the Tertiary conglomerates of Upper Oligocene age that are common along the eastern borders of the Ebro basin. The site location is privileged as far as mobility and resource distribution are concerned. On the one hand, the Francolí valley is a natural passage linking the Ebro basin and the coastal Mediterranean regions. On the other hand, it is an ecotone between the plain of the Ebro basin and the mountain domain of the Prades Mountains, 4 km south of the site. In addition, good-quality flint outcrops are common in the region. Although the Molí del Salt was reported as an archeological site in the 1950s [1], the first excavations were carried out in 1999 and consisted of a test pit of 3 m^2 that allowed documenting of the whole stratigraphic sequence [2]. When the excavations started, the rockshelter was entirely filled by sediments, and most of the roof was collapsed. After the positive results yielded by the exploratory works, a research project was undertaken in 2001 and is still in progress under the direction of one of us (M.V.). The excavated area has been extended from the first excavations up to 70 m^2 of the current excavation area.

The results from the research carried out since 1999 has been extensively presented in different publications [2-6]. Therefore, here, we will only present the basic data on the Molí del Salt chronology and archeological record. The stratigraphic sequence is 2.5 m thick (Fig A) and contains Mesolithic (level Sup) and Late Upper Paleolithic (Late Magdalenian) layers (units A and B). Seventeen ^{14}C/AMS dates have

been obtained (Table 1). For unit B, there are three dates available that situate this unit between 15,300 and 13,498 cal years BP. Unit A shows a chronological range between 13,800 and 12,670 cal years BP. These results situate the late Upper Paleolithic layers (units A and B) at the end of the Pleistocene. The Mesolithic of level Sup, according to the radiocarbon date available, which indicates a chronological span between 9110 and 8710 cal BP, would correspond to the beginning of the Holocene. This date is consistent with the overall chronology of Mesolithic sites in the Iberian Peninsula. Altogether, the collapse of the rockshelter ceiling would be situated around the Pleistocene–Holocene boundary.

Table 1. ^{14}C AMS dates from Molí del Salt. ^{14}C ages have been converted to calendar ages by using the CalPal2007-HULU Calibration Curve in the CalPal calibration software [7].

Level	Ref. lab.	Material	^{14}C age	Years cal BP (2σ)	Years cal BC (2σ)
Sup	Beta-173335	Bone	8040 ± 40	9110-8710	7160-6760
Asup	Beta-179599	Charcoal	10,840 ± 50	12,890-12,690	10,940-10,740
Asup	Beta-179598	Charcoal	10,990 ± 50	13,050-12,730	11,100-10,780
Asup	Beta-221912	Charcoal	11,060 ± 70	13,130-12,770	11,180-10,820
Asup	Beta-221913	Charcoal	10,850 ± 70	12,950-12,670	11,000-10,720
Asup	Beta-235268	Charcoal	10,920 ± 60	12,990-12,710	11,040-10,760
A	Beta-367195	Charcoal	10,940 ± 50	13,059-12,695	11,109-10,745
A	Beta-235267	Charcoal	11,000 ± 60	13,080-12,720	11,130-10,770
A	Beta-367196	Charcoal	11,090 ± 50	13,188-12,772	11,238-10,822
A	Beta-277000	Charcoal	11,230 ± 50	13,270-13,030	11,320-11,080
A	Beta-277001	Charcoal	11,440 ± 60	13,500-13,180	11,550-11,230
A	Beta-284214	Charcoal	10,940 ± 50	13,059-12,695	11,109-10,745
A	Beta-284212	Charcoal	11,770 ± 50	13,790-13,550	11,840-11,600
A	Beta-284213	Charcoal	11,800 ± 50	13,800-13,560	11,850-11,610
B1	Beta-367197	Bone	11,880 ± 50	14,062-13,498	12,112-11,548
B1	GifA-101037	Charcoal	11,940 ± 100	14,070-13,590	12,120-11,640
B2	GifA-101038	Charcoal	12,510 ± 100	15,300-14,540	13,350-12,590

The excavation methodology is based on a grid of 1x1-m squares. Archeological remains larger than 1 cm are mapped in three dimensions. All of the excavated sediment is wet sieved through a 1-mm mesh, allowing the recovery of an enormous quantity of micro remains. The Molí del Salt excavations have yielded an abundant archeological record, mostly made up of thousands of lithic and faunal remains. In addition, three

human teeth corresponding to an infant individual were recovered in level A. The identified animal and botanical resources indicate the exploitation of the surrounding environment of the site within the context of a broad-spectrum economy. The faunal assemblage is typical of the Late Upper Paleolithic sites from Mediterranean. Rabbit (*Oryctolagus cuniculus*) is the most represented species at every level of Molí del Salt [8, 9], accounting for more than 90% of the bones identified. Other documented species are: ibex (*Capra pyrenaica*), red deer (*Cervus elaphus*), wild boar (*Sus scropha*), lynx (*Lynx pardina*), fox (*Vulpes vulpes*), and badger (*Meles meles*). Some bird remains, most of them from the Perdicinae subfamily, have also been identified, particularly at the bottom of the sequence (level B2).

The zooarcheological analysis indicates that all of the anatomical parts of rabbits are represented in the faunal assemblage, which suggests that these animals were transported whole to the site, although some skeletal parts, such as vertebrae and ribs, are comparatively less represented [10]. Most of the bones, especially long limb bones, appear fractured on both edges. This breakage pattern is typical of human consumption, which implies that rabbits were carried and consumed by humans rather than being the result of natural transportation or another predator's action. Moreover, cut-marks on different anatomical parts support the anthropogenic character of the bone assemblage and correspond to different stages of faunal processing (skinning, defleshing, and disarticulation). Macromammals show a much more selective introduction. Not all of the skeletal parts are present, which means their carcasses were not entirely transported to the site. Ibex and red deer remains exhibit a dominance of limb bones, whereas axial and cranial parts are underrepresented. Likewise, in the case of rabbit, cut-marks and anthropic breakage indicate human activity. However, it cannot be ruled out that some of these remains were transported to the site as raw material to make artefacts. Nearly

50 bone artefacts have so far been recovered. There are also bone fragments that seem to correspond to waste products from the fabrication process as well as unfinished artefacts.

The study of wood charcoal from units A and B has allowed the identification of the following taxa: *Pinus sylvestris*, *Juniperus*, *Acer*, *Betula*, *Corylus avellana*, *Prunus*, *Rhamnus cathartica*/*saxatilis*, Rosaceae/Maloideae, and *Sambucus*. Apart from the use of wood as fuel, it cannot be ruled out that some of the identified species had other uses, such as fruit consumption. This consumption of wild fruits would be supported by the finding of charred seeds. The identified taxa are blackthorn (*Prunus spinosa*), hawthorn (*Crataegus*) and rose hip (cf. *Rosa*) [8]. This vegetal spectrum suggests that the climatic conditions were slightly colder and drier that the current ones, but the improvement characteristic of the Tardiglacial is also evident. This is consistent with the radiocarbon dates, which indicate a Greenland Interstadial 1 (GI-1) chronology for most of the sequence.

Although subsistence was mainly based on local resources, the marine shells found at both the Pleistocene and the Holocene layers show a major mobility range. Different shellfish species have been documented (*Pecten jacobaeus*, *Glycymeris glycymeris*, *Cyclope* sp., *Dentalium* sp.), which suggests either movements up to the coastline (currently situated approximately 36 km away in a straight line), or contacts with populations living closer to the Mediterranean Sea. Most of these shells are perforated, which indicates that they were used as pendants or beads.

The study of the lithic assemblage indicates that flint was clearly the dominant raw material in tool production. This raw material is particularly abundant in the region, and different flint sources have been identified in a 20-km radius around the site. Most of the flint artifacts found in Molí del Salt exhibit the typical characteristics of these

regional outcrops [10]. Nevertheless, there are other varieties from outcrops outside the region. Along the archaeological sequence, some evolutionary trends in raw material provisioning suggest significant modifications in the mobility strategies. Exogenous flint shows higher percentages at the earliest levels (unit B), but its representation decreases toward the upper part of the sequence. This tendency culminates at the Mesolithic level, when procurement strategies show a more local character. These changes can be interpreted within the context of the reduction of exploitation territories as long as we approximate to the end of the Paleolithic. Other raw materials, such as schist, limestone, and sandstone, were also occasionally used for knapping. Unworked cobbles of limestone, schist and granite were mainly used as hammerstones, anvils, and working surfaces. In addition, limestone cobbles and schist slabs were used as supports for engravings. These raw materials are abundant in the colluvial and alluvial deposits close to the site and can be considered strictly local.

Different knapping methods were used for tool production [11, 12]. Blade production is well represented, although it is not dominant in any level. Cores for systematic blade debitage are uncommon, and the blade percentage represents, in general, 20% of blanks. However, many cores show a tendency to produce elongated flakes. Often, these products are obtained from unipolar or bipolar cores with hierarchical structures that are characterized by a well-defined flaking surface opposite another that is not knapped. In addition, more expedient knapping methods, oriented to the production of short flakes from discoidal or polyhedral cores, have also been documented. Cores are generally small and indicate a systematic detachment of small products, at least in the last stage of the reduction sequence.

Endscrapers are the best represented tool group in the Upper Paleolithic layers, especially at unit A, where they reach approximately 40% (Fig C). Other tool types

show lower percentages but are well represented, such as backed elements (points and blades), denticulates, truncations, burins, and borers. However, there are significant changes through the sequence, emphasizing the differences between units B and A already detected in raw material procurement. Unit B is characterized by the high percentage of truncations (27% and 22% at levels B2 and B1, respectively). The toolkit is also composed of endscrapers (20% and 19%), denticulates (12% and 16%), and backed artefacts (13% and 12%). The absence of burins at these lower levels should be highlighted. Unit A, in contrast, shows an abrupt decrease of truncations that are reduced to values of approximately 5%, while endscrapers increase until their maximum value. Denticulates and backed artefacts do not show significant changes. Burins increase significantly with regard to unit B, with a maximum of 10% in unit A. The typological pattern of unit A is typical of Late Upper Paleolithic assemblages from the Mediterranean basin of the Iberian Peninsula. The dominance of truncations characteristic of unit B is less common, although it has also been documented in other Magdalenian sites. The Mesolithic assemblage of level Sup shows a clear break with these typological trends, and it is characterized by the dominance of denticulates and notches.

Use-wear analysis has confirmed that endscrapers were specially used for hide working [13]. Nevertheless, endscrapers were not the only tool used in hide working because other tools, such as truncations, denticulates, and unretouched flakes, also exhibit the use-wear associated with such activity. Backed elements were used as projectile points, and defleshing use-wear has been identified mainly in unretouched flakes. Recycling and reusing of artefacts have been documented by identifying burned tools that were retouched after the heat damage. It has been calculated [14] that at least 7.5% of the retouched artefacts were made on recycled blanks. Recycling is mainly

associated with artefacts used in domestic contexts (endscrapers, denticulates, borers), which demonstrates its expedient character and its capacity to attend to immediate needs within the context of daily activities.

In general, the archeological record is consistent with the interpretation of Molí del Salt as a residential site. Both the faunal and the lithic assemblages indicate that domestic activities were commonly carried out. Faunal resources (especially rabbits) were brought to the site, processed and consumed. Knapping is also well attested, and the tool variability suggests that a wide array of activities were conducted in the rockshelter, including some largely domestic tasks. Among these, hide-working is particularly significant because use-wear analysis indicates that both fresh and dry hide was processed at the site. The manufacture of bone tools and colorant processing has also been documented. Combustion evidence is also common, as shown by the high percentages of bones and lithics with thermal damage. Although discrete hearths are difficult to identify due to palimpsest dynamics, several combustion features have been documented, demostrating a high degree of variability in hearth construction [15].

Mobiliary art

The discovery of portable art pieces has been one of the most outstanding aspects of the archaeological record from Molí del Salt [16], particularly in the context of Northeastern Iberia, where Upper Paleolithic art has so far been scarcely documented. Thirteen pieces of mobiliary art with engravings have been found so far, including the piece discussed in this paper. Nine of them are schist slabs, three are limestone cobbles, and the remaining piece is a bone fragment. Both schist and limestone are abundant in the surroundings of the site. Schist comes from the Paleozoic

formations of the Prades Mountains, 4 km south of the site, and slabs are abundant in the alluvial deposits of the Milans River in front of the rockshelter. Limestone cobbles are overwhelmingly dominant in the conglomerates forming the rockshelter. Schist slabs used as blanks are larger and can reach 30 cm long, while the engraved limestone pebbles are 6-7 cm. Because they are larger and offer plane surfaces that are suitable as graphic spaces, schist slabs show more complex representations, combining several figures on one or two surfaces. Except for two slabs (one out of stratigraphic context and another found in an uncertain context), the rest of the evidence has a clear stratigraphic attribution. Most of the pieces correspond to Unit A, except the slab presented here, which comes from the top of Unit B. Unlike Unit B, the engraved slabs from unit A are on quartzitic schist, which is harder than the grauvaquic schist.

Eight pieces show figurative representations, either exclusively or in combination with schematic motifs. In total, 18 figures have been recognized, most of them corresponding to undetermined animals, although it has been possible to identify depictions of cervids, horses and bovines, as well as one human figure. Representations were made with fine incision. Some animal figures are incomplete, and there is no interest in representing anatomical internal details, even though in one case the infilling of the internal part of the animal figure with transversal lines has been documented. From a stylistic point of view, the slenderness of figures from unit A is remarkable. This stylization trend can be observed in limb, trunk, and especially neck enlargements, and it is typical of the late Upper Paleolithic art of Mediterranean Iberia.

References

1. Vilaseca S. Las industrias del sílex tarraconenses. Madrid: Consejo Superior de Investigaciones Científicas; 1954.

2. Vaquero M. Els darrers caçadors-recol·lectors de la Conca de Barberà: el jaciment del Molí del Salt (Vimbodí). Excavacions 1999-2003. Montblanc: Museu-Arxiu de Montblanc i Comarca; 2004.
3. García Díez M, Martin i Uixan J, Gené JM, Vaquero M. La plaqueta gravada del Molí del Salt (Vimbodí, Conca de Barberà) i el grafisme paleolític/epipaleolític a Catalunya. Cypsela 2003; 14: 159-173.
4. Angelucci D, Gené JM, Ollé A, Vaquero M, Vergés JM, Allué E et al. Darreres intervencions arqueològiques en jaciments paleolítics de la Conca del Francolí: la Cansaladeta (la Riba, Alt Camp) i el Molí del Salt (Vimbodí, Conca de Barberà). Tribuna d'Arqueologia 2003; 1999-2000: 23-63.
5. Vaquero M, Allué E, Alonso S, Campeny G, Estrada A, García M et al. Una nueva secuencia del Paleolítico Superior final en el Sur de Cataluña: el Molí del Salt (Vimbodí, Tarragona). In: Bicho NF, editor. O Paleolítico. Actas do IV Congresso de Arqueologia Peninsular (Faro, 14 a 19 de Setembro de 2004). Faro: Universidade do Algarve; 2005. pp. 493-508.
6. Vaquero M, Alonso S. El Molí del Salt. In: Sala R, editor. Pleistocene and Holocene Hunter-Gatherers in Iberia and the Gibraltar Strait: the Current Archaeological Record. Burgos: Universidad de Burgos & Fundación Atapuerca; 2014. pp. 295-302.
7. Weninger B, Jöris O. A 14C age calibration curve for the last 60 ka: the Greenland-Hulu U/Th timescale and its impact on understanding the Middle to Upper Paleolithic transition in Western Eurasia. Journal of Human Evolution 2008; 55: 772-781.

8. Allué E, Ibáñez N, Saladié P, Vaquero M. Small preys and plant exploitation by late pleistocene hunter-gatherers. A case study from the Northeast of the Iberian Peninsula. Archaeological and Anthropological Sciences 2010; 2: 11-24.
9. Ibáñez N, Saladié P. Acquisition anthropique d'*Oryctolagus cuniculus* dans le site du Molí del Salt (Catalogne, Spagne). In: Brugal J-Ph, Desse J, editors. Petits animaux et sociétés humaines. Du complément alimentaire aux ressources utilitaires. XXIVe Rencontres Internationales d'Archéologie et d'Histoire d'Antibes. Sophia Antipolis: Éditions APDCA; 2004. pp. 255-259.
10. Soto M, Gómez de Soler B, Vallverdú J, Vaquero M. Potential siliceous sources during Prehistory: Results of prospecting in the East margin of the Ebro basin (NE Iberian Peninsula). Journal of Lithic Studies 2014; 1: 293-318.
11. Vaquero M, Gené JM, Alonso S. La indústria lítica. In: Vaquero M, editor. Els darrers caçadors-recol·lectors de la Conca de Barberà: el jaciment del Molí del Salt (Vimbodí). Montblanc: Museu-Arxiu de Montblanc i Comarca; 2004. pp. 69-134.
12. García Catalán S, Gómez de Soler B, Soto M, Vaquero M. Los sistemas de producción lítica en el Paleolítico Superior final: el caso del nivel Asup del Molí del Salt (Vimbodí i Poblet, Tarragona). Zephyrus 2013; LXXII: 39-58.
13. Martínez Molina K. Anàlisi funcional del artefactes lítics. In: Vaquero M, editor. Els darrers caçadors-recol·lectors de la Conca de Barberà: el jaciment del Molí del Salt (Vimbodí). Montblanc: Museu-Arxiu de Montblanc i Comarca; 2004. pp. 135-150.
14. Vaquero M, Alonso S, García-Catalán S, García-Hernández A, Gómez de Soler B, Rettig D et al. Temporal nature and recycling of Upper Paleolithic artifacts: the burned tools from the Molí del Salt site (Vimbodí i Poblet, northeastern Spain). J Archaeol Sci. 2012; 39: 2785–2796.

15. Vallverdú J, Gómez de Soler B, Vaquero M. L’organització de l’espai. In: Vaquero M, editor. Els darrers caçadors-recol·lectors de la Conca de Barberà: el jaciment del Molí del Salt (Vimbodí). Montblanc: Museu-Arxiu de Montblanc i Comarca; 2004. pp. 201-210.
16. García Díez M, Vaquero M. La variabilité graphique du Molí del Salt (Vimbodí, Catalogne, Espagne) et l'art mobilier de la fin du Paléolithique supérieur à l'est de la Péninsule Ibérique. L’Anthropologie. 2006; 110: 453-481.
17. Vallverdú J, Carrancho A. Estratigrafia del Molí del Salt. In: Vaquero M, editor. Els darrers caçadors-recol·lectors de la Conca de Barberà: el jaciment del Molí del Salt (Vimbodí). Montblanc: Museu-Arxiu de Montblanc i Comarca; 2004. pp. 61-68.

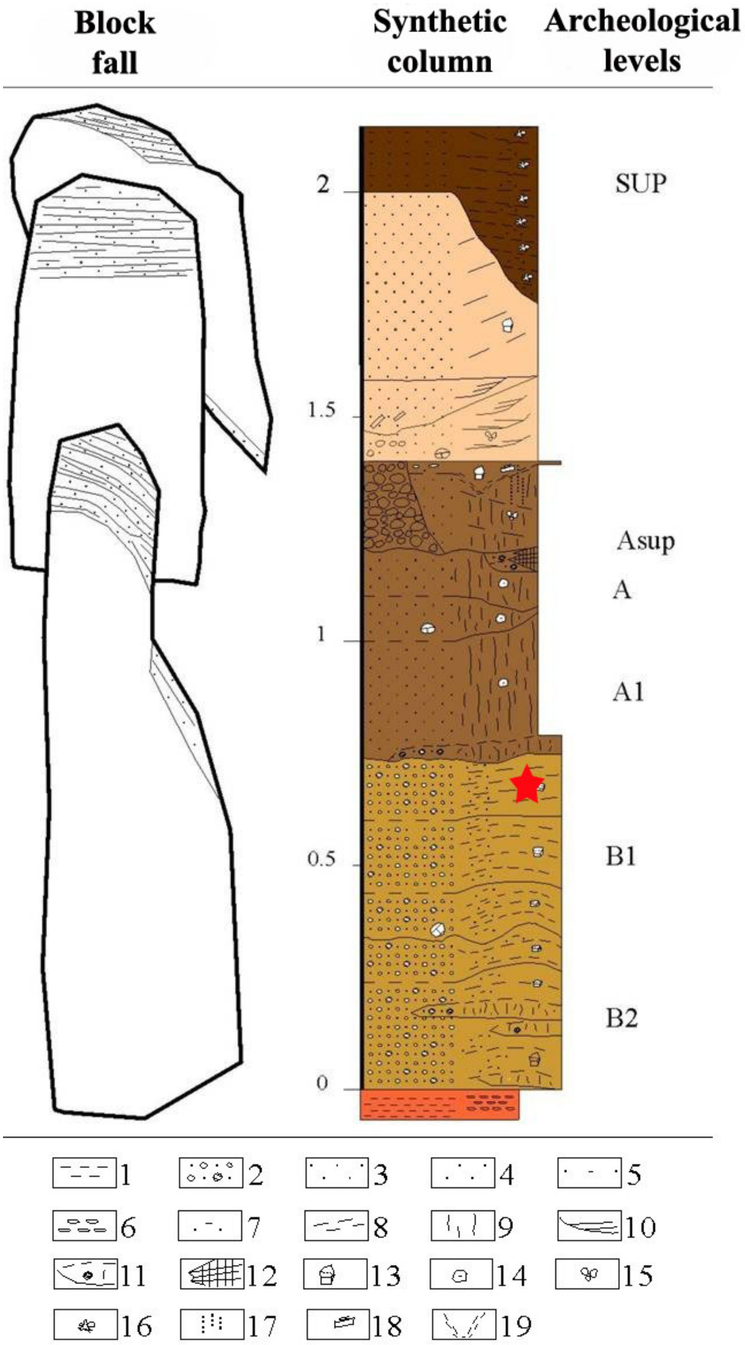

Fig A. Stratigraphic sequence of Molí del Salt, according to Vallverdú and Carrancho [17]. Lithology: 1. Clay. 2. Gravel. 3. Silt. 4. Sandstone. 5. Sand-Clay. Sedimentary structure: 6. Nodular. 7. Graded. 8. Plane not paralleled. 9. Massive. 10. Channel. 11. Lenticular massive. 12. Hearth. Edaphic traits: 13. Polyhedron. 14. Microaggregates. 15. Particular. 16. Crumbly rock. 17. Pseudomycelia. 18. Calcitic *pendents*. The red star indicates the stratigraphic location of the engraved slab.

A

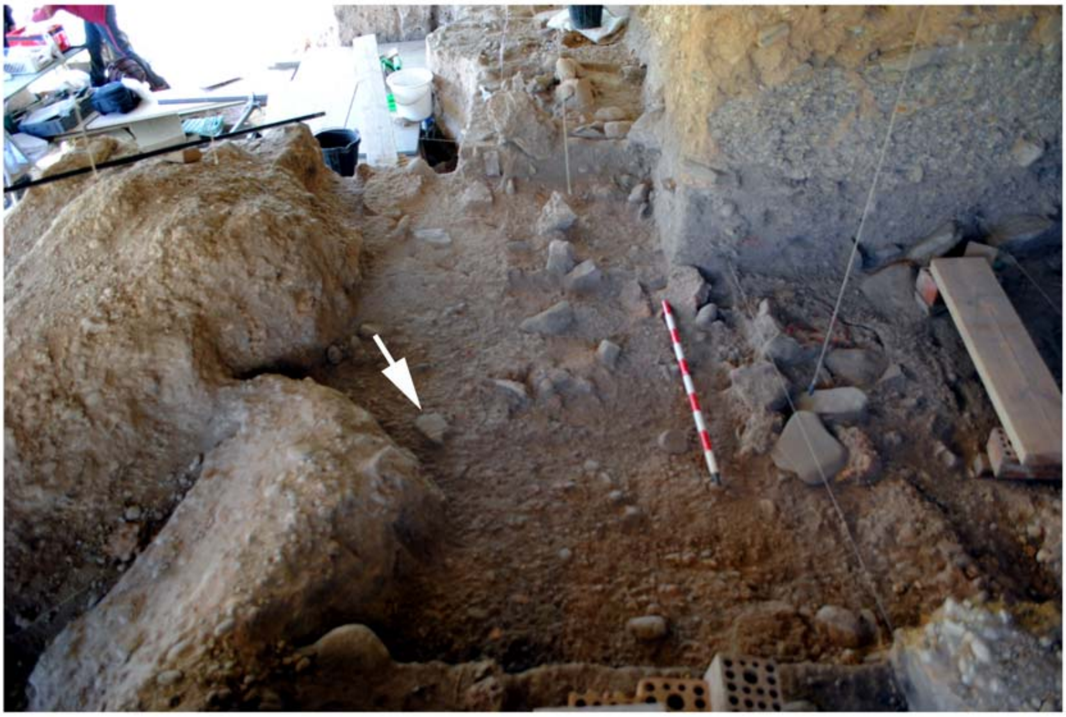

B

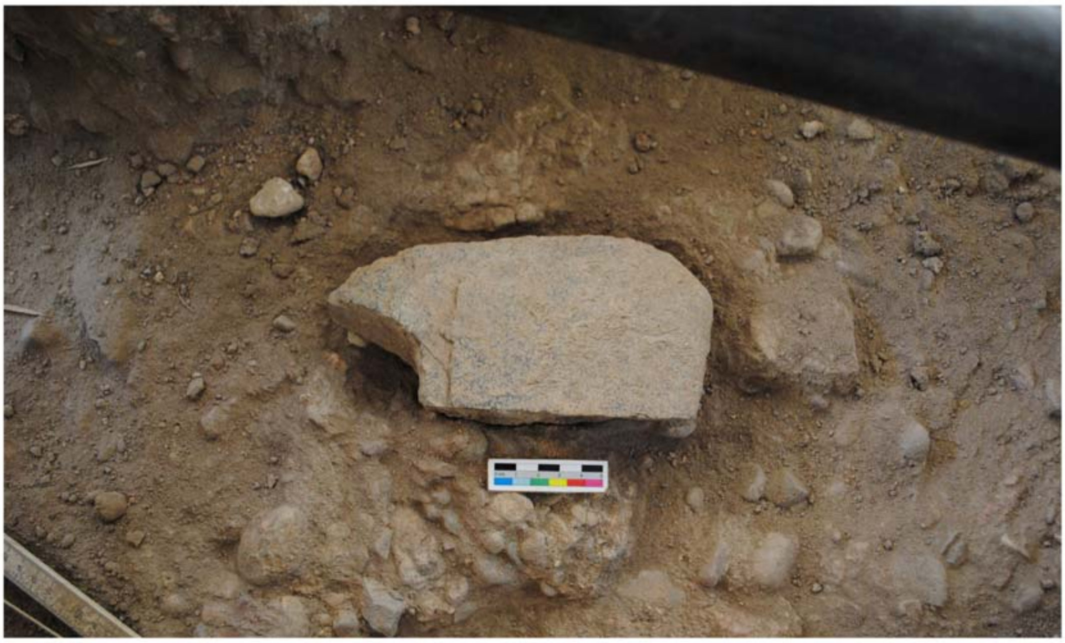

Fig B. A. View of the excavation surface corresponding to the upper part of level B1. The arrow indicates the location of the engraved slab. B. *In situ* image of the engraved slab.

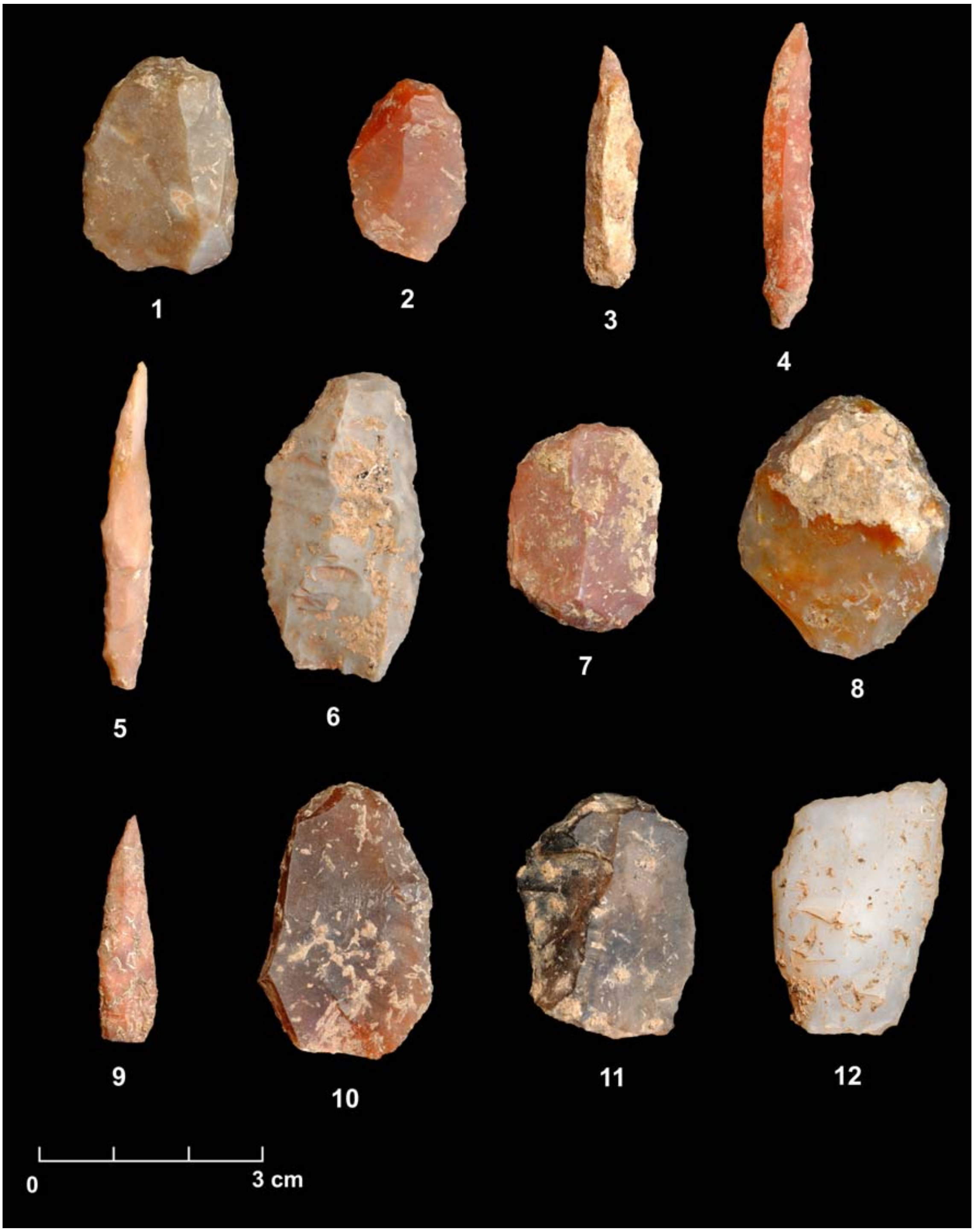

Fig C. Lithic tools from the late Magdalenian assemblages of Molí del Salt: endscrapers (1, 2, 6-8, 10 and 11), backed elements (3-5 and 9), and truncation (12).
