# Supplementary material for: Looking at the Camp: Paleolithic Depiction of a Hunter-Gatherer Campsite
Source: PLoS One. 2015 Dec 2;10(12):e0143002. doi: 10.1371/journal.pone.0143002 (PMC4668041; doi:10.1371/journal.pone.0143002)
Supplement: S2 File — Individualized description of the graphic units. (PDF) [file pone.0143002.s003.pdf]

S2 File. Individualized description of the graphic units

Graphic unit A (Fig B). This unit is located in the lower left corner. Its morphology tends to be semicircular and has been shaped by a straight line at the bottom and another, convex line to define the closed character. The outline of the convex line tends to be defined by the bottom line except on its right side, where the stroke goes beyond the bottom line. The bottom line exceeds the limit defined by the curved line, continuing with a delineation that is slightly concave on the right side and angular on the left side. Inside the closed space, at least eight lines were obliquely drawn (two possible short lines are documented in the bottom right area, although we are unsure about their anthropic character due to their very superficial character and the taphonomic problems). These oblique lines traverse most of the interior space, although only one of them touches the curved contour line. The maximum dimensions of the unit are 43 mm wide and 22 mm high; the maximum dimensions of the semicircular structure (excluding the appendices of the lower contour line) are 30 mm wide and 22 mm high. The technique of execution is incised engraving of a simple contour. The grooves have a U section and a medium and superficial (the grooves on the bottom left) depth. Most of the lines correspond to a single stroke, except for the left and lower contour lines as well as possibly the second (from left to right) inner line. We have documented some corrections aimed at changing the line layout, either to modify – mainly to curve – its direction (left end of the lower contour line and distal end of the curved contour line) or to slightly divert it (proximal ends of the curved contour line). The direction of the strokes is from top to bottom (right side of the curved contour line and two inner lines), from bottom to top (left side of the curved contour line, left side of the bottom line and four inner contour lines), from left to right (one of the strokes of the lower contour line), from right to left (right-left distal end of the curved line and two

inner contour lines) and bi-directional (lower contour line). Line overlapping shows that one inner line was made after the curved line boundary, the lower contour line is prior to the prolongation line of the right end of the curved contour line, and the right side of the curved contour line is before the distal configuration of this same line.

Graphic Unit B (Fig C). This unit is located at the bottom center area. Its morphology tends to be semicircular due to the combination of one straight line at the bottom and a convex line that defines the closed character of the unit and whose outline is defined by the bottom line. The bottom line exceeds the limit defined by the curved line, continuing slightly curved toward the left side and straight toward the right side. The closed area is filled with 12 horizontal lines that run along most of the interior space, although only one touches the curved contour line. The maximum dimensions are 31 mm wide and 18 mm high; the maximum dimensions of the semicircular structure (excluding the appendices of the lower contour line) are 20 mm wide and 18 mm high. The technique of execution is incised engraving and a simple outline form. The grooves have a U section and medium and shallow depth (although less so in the distal ends of the lines, the shortest inner line, and the left side of the curved contour line). Most of the lines correspond to a single stroke, except the convex contour line, two inner lines, and the lower contour line. The left end of the lower contour line shows corrections aimed at defining its outline. The direction of the strokes is from top to bottom (right side of the curved contour line), from bottom to top (left side of the curved contour line), from right to left (lower contour line, distal end of the curved contour line, and five interior lines), and bi-directional left-right (two inner lines). Line overlapping shows that the distal stroke of the curved contour line is earlier than the right side of the curved contour line, one inner line is later that the curved contour line, the bottom contour line is earlier than the small strokes on its left end, the right side of the bottom contour line was made

after the center of the line, and the left side of one of the inner lines is posterior to the right side.

Graphic Unit C (Fig D). This unit is located in the lower right corner. Its shape tends to be semicircular, and it is defined by a straight line at the bottom and a convex line that creates the closed character of the motif and whose outline tends to be defined by the bottom line. A long vertical line (33 mm) starts from the top of the convex line. Inside the closed space, seven oblique (although nearly vertical) lines were drawn that run along most of the interior space but do not touch the curved contour line. The closed structure has maximum dimensions of 20 mm wide and 15 mm high; the maximum dimensions of the semicircular structure (excluding the appendices of the lower contour line) are 18 mm wide and 15 mm high. The technique of execution is incised engraving with a simple outline. The grooves have a U section and medium or shallow depth. Most of the lines correspond to a single stroke, except the curved contour line and the straight line representing the upper appendix. The distal end of the curved contour line exhibits a small rectification aimed at defining the curved character of the line. The direction of the strokes is from top to bottom (right side of the curved contour line and one inner line) and bi-directional left-right (one inner line). Line overlapping shows that the left side of the curved line is after the right side of the same line.

Graphic unit D (Fig E). This unit is located in the upper left corner. Its morphology tends to be semicircular and is made up of a straight line at the bottom and a curved line that determines the closed character and whose outline tends to be defined by the bottom line. This bottom line presents a double stroke in the right-center sector and exceeds the limit defined by the curved line, continuing straight on the left side. Inside the closed space, at least eight horizontal lines were drawn. These lines cover most of the inner area (especially in the center and the left side), but only two of them

touch the curved contour line. The maximum dimensions are 25 mm wide and 14 mm high; the maximum dimensions of the semicircular structure (excluding the appendices of the lower contour line) are 22 mm wide and 14 mm high. The technique of execution is incised engraving with a simple outline. The grooves have a U section and a mainly superficial or medium depth. Most lines correspond to more than one stroke: at least five in the curved contour line, at least two in the bottom line, and at least two in several of the interior lines. The direction of the strokes is from top to bottom (left and right sides of the curved contour line), from left to right (lower stroke of the bottom line and two inner lines), and from right to left (two inner lines). Line overlapping shows that one inner line is after the curved contour line, and on the left side of the curved line, the upper stroke is earlier than the lower one.

Graphic unit E (Fig F). This unit is located in the middle-left part of the upper sector. Its shape tends to be semicircular, and it is defined by a straight line at the bottom and a convex line that creates the closed character of the motif and whose outline tends to be defined by the bottom line. The bottom line exceeds the limit defined by the curved line, continuing curved toward the left and straight toward the right side. At least 10 oblique lines were drawn inside the closed space. They cover most of the interior area (especially in the central and right side), and only three of them touch the curved contour line. The maximum dimensions are 32 mm wide and 19 mm high; the maximum dimensions of the semicircular structure (excluding the appendices of the lower contour line) are 26 mm wide and 19 mm high. The technique of execution is the incised engraving with a simple outline. The grooves have a U section and medium and shallow (especially the interior lines) depth. Most of the lines were made with a single stroke, except for the curved line and one interior line. The direction of the strokes is from top to bottom (right side of the curved contour line and internal lines), from the

bottom up (one of the strokes in the left side of the curved contour line), from right to left (bottom contour line), and bi-directional top-down (the other stroke in the left side of the curved contour line). Line overlapping shows that the inner lines were drawn after the curved contour line, the uppermost stroke in right end of the bottom contour line is older than the lower one, the lowermost line in the right side of the curved contour line is after the upper line, and the shortest line in the upper left side of the curved contour line is earlier than the longest stroke mainly defining the curved outline.

Graphic unit F (Fig G). This unit is located in the medial-right part of the upper sector. Its shape tends to be semicircular, and it is defined by a straight line at the bottom and a convex line that creates the closed character of the motif and whose outline tends to be defined by the bottom line. The bottom line exceeds the limit defined by the curved line, continuing straight on the right side. At least 11 oblique lines (although with a horizontal trend) were drawn inside the closed space, covering most of this interior area (especially in the central and right side). Only one of them touches the curved contour line. The maximum dimensions are 34 mm wide and 18 mm high; the maximum dimensions of the semicircular structure (excluding the appendices of the bottom contour line) are 25 mm wide and 18 mm high. The technique of execution is incised engraving with a simple outline. The grooves have a U section and medium and shallow (particularly the inner lines) depth. Most of the lines correspond to a single stroke, except for the curved contour line. The left side of the bottom contour line shows a small correction leading to a slight diversion of the line. The direction of the strokes is from top to bottom (the two sectors of the curved contour line), from right to left (two inner lines), and from left to right (bottom contour line and one inner line). Line overlapping shows that the curved contour line was drawn starting from the left toward the right side.

Graphic unit G (Fig H). This unit is located in the upper right sector. Its morphology tends to be semicircular and is made up of a straight line at the bottom and a curved line that determines the closed character and whose outline tends to be defined by the bottom line. Most of the straight bottom line shows a double stroke and exceeds the limit defined by the curved line, continuing straight to the left and curved to the right. At least 11 oblique lines were drawn inside the closed space, covering most of this interior area. They do not touch the curved contour line, although two of them touch the bottom contour line. The maximum dimensions are 33 mm wide and 19 mm high; the maximum dimensions of the semicircular structure (excluding the appendices of the lower contour line) are 26 mm wide and 19 mm high. The technique of execution is incised engraving with a simple outline. The grooves have a U-section and medium and shallow depth. Most of the interior lines exhibit parasitic grooves. Most lines correspond to a single stroke, except for the curved line and the bottom contour line. In some sectors, there are corrections aimed at changing the outline, either for modifying the direction of the line (right end of the bottom contour line) or deflecting it slightly (left end of the bottom contour line). The bottom contour line has a greater correction or reinforcement because it is partly drawn with a double stroke. The direction of the strokes is from bottom to top (left side of the curved contour line) and from right to left (lower contour line and two inner lines). Line overlapping shows that the bottom contour line is after the curved contour line, the rectification of the left end of the bottom line was made after the contour lines, the line for strengthening the lower contour line is later than the general layout of this line, and one interior line is after the bottom contour line.

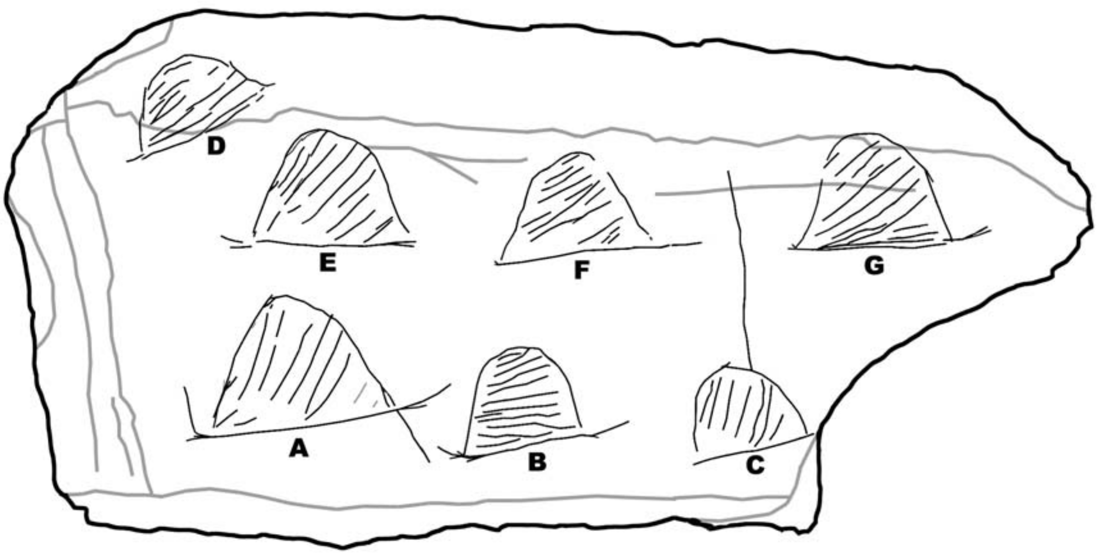

Fig A. Drawing of the engraving with the letter corresponding to each graphic unit.

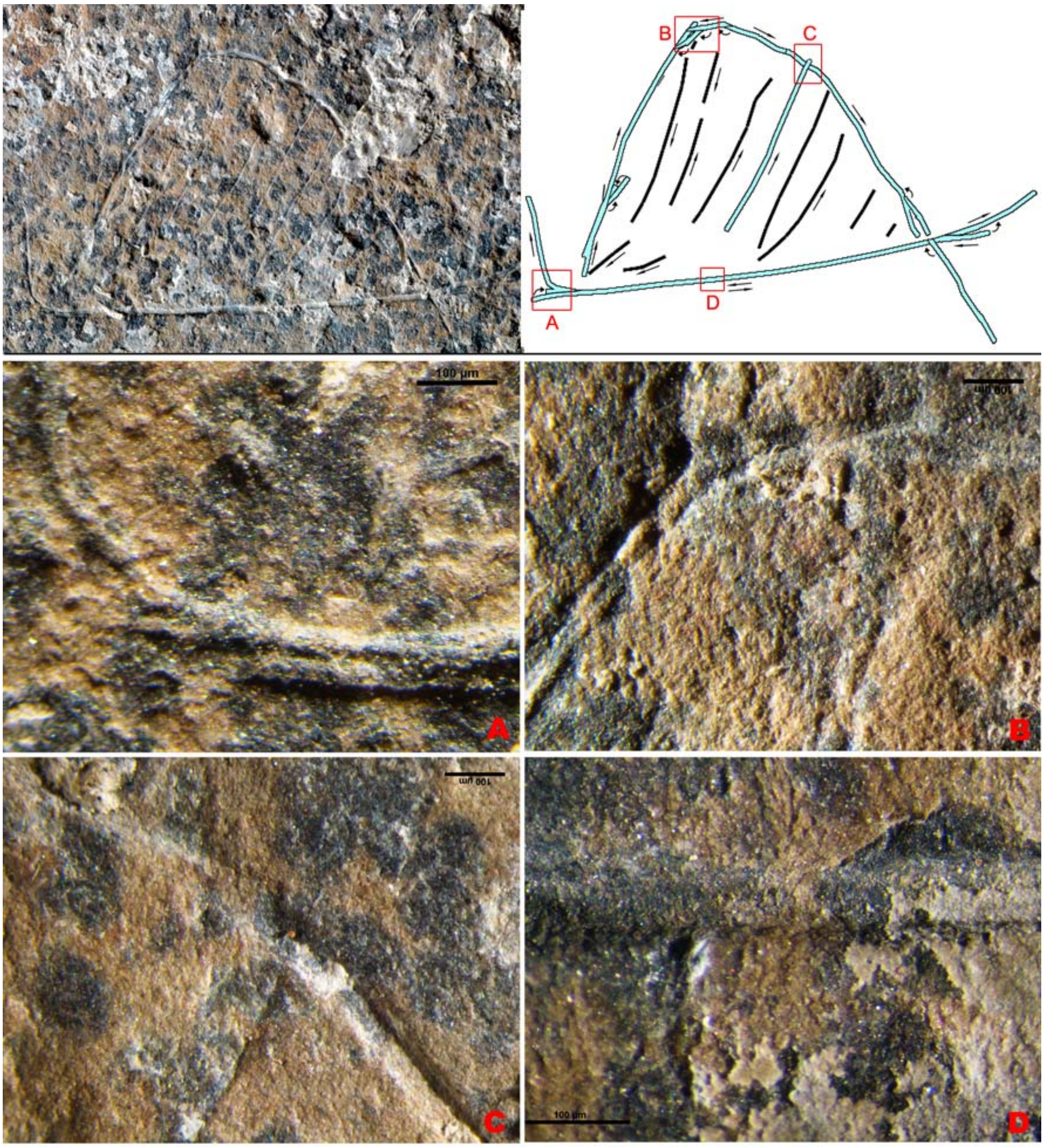

Fig B. Engraving process of the graphic unit A.

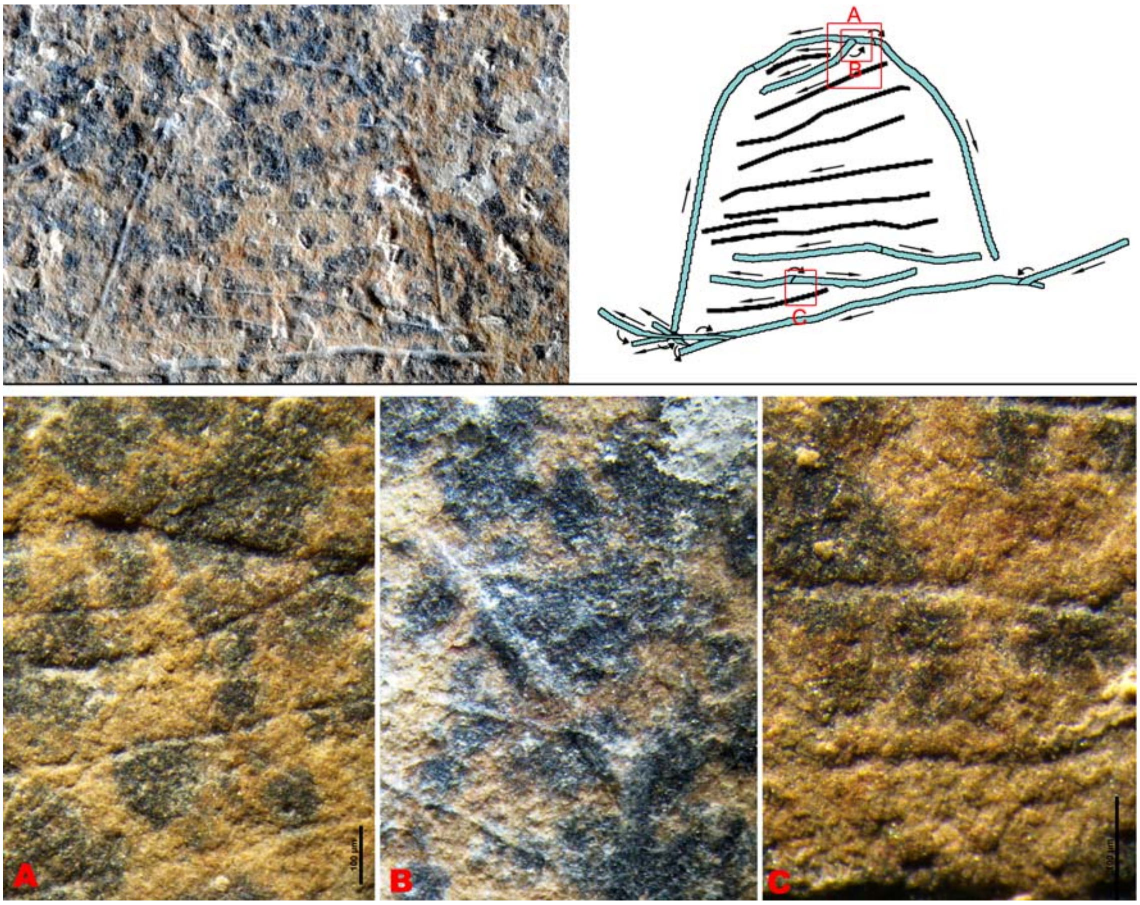

Fig C. Engraving process of the graphic unit B.

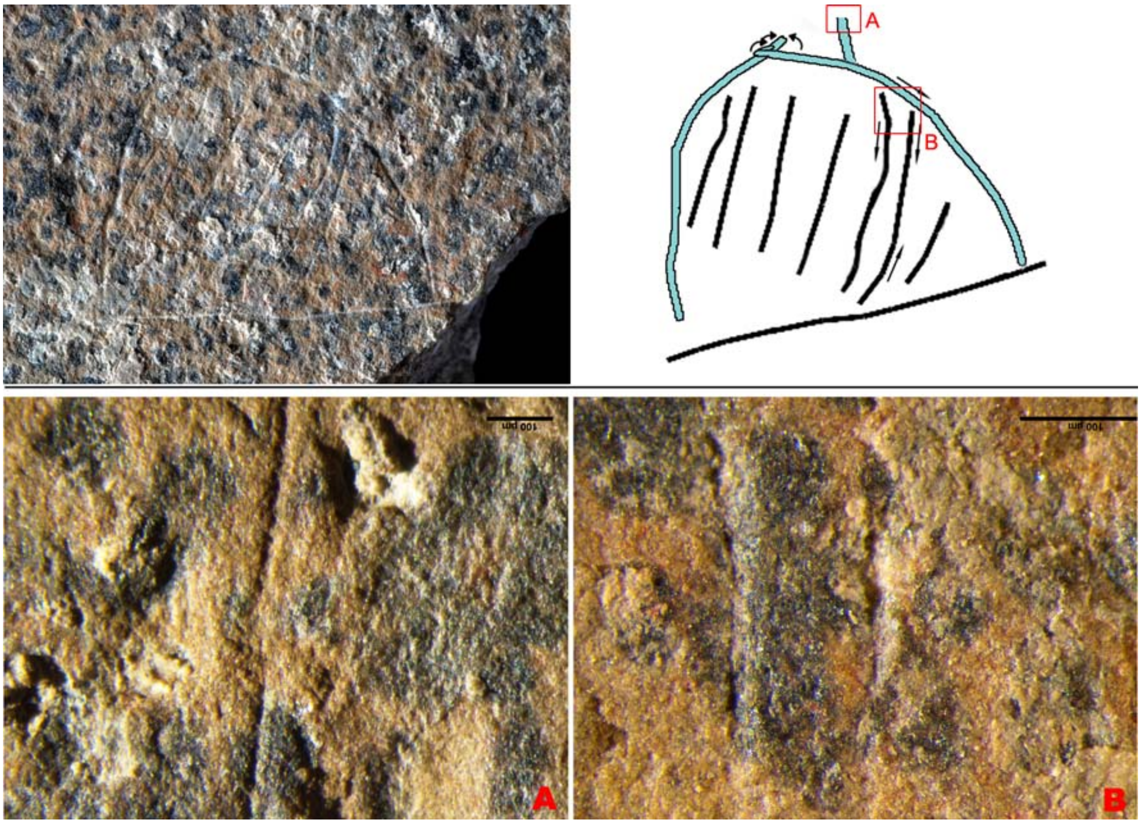

Fig D. Engraving process of the graphic unit C.

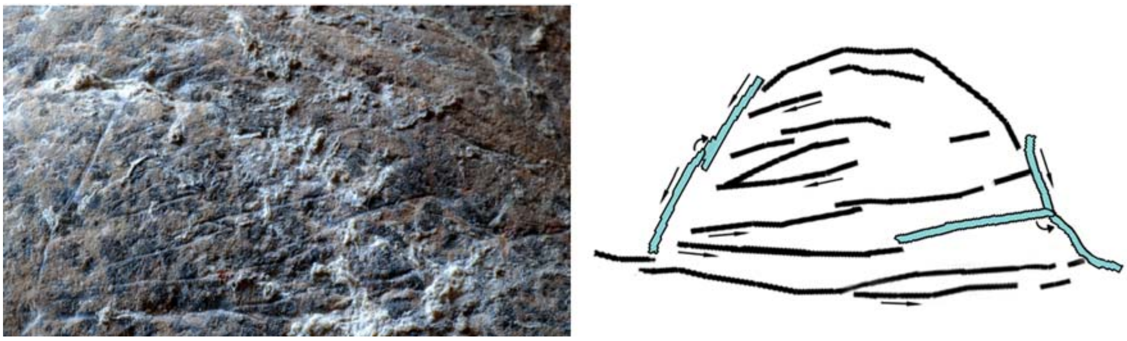

Fig E. Engraving process of the graphic unit D.

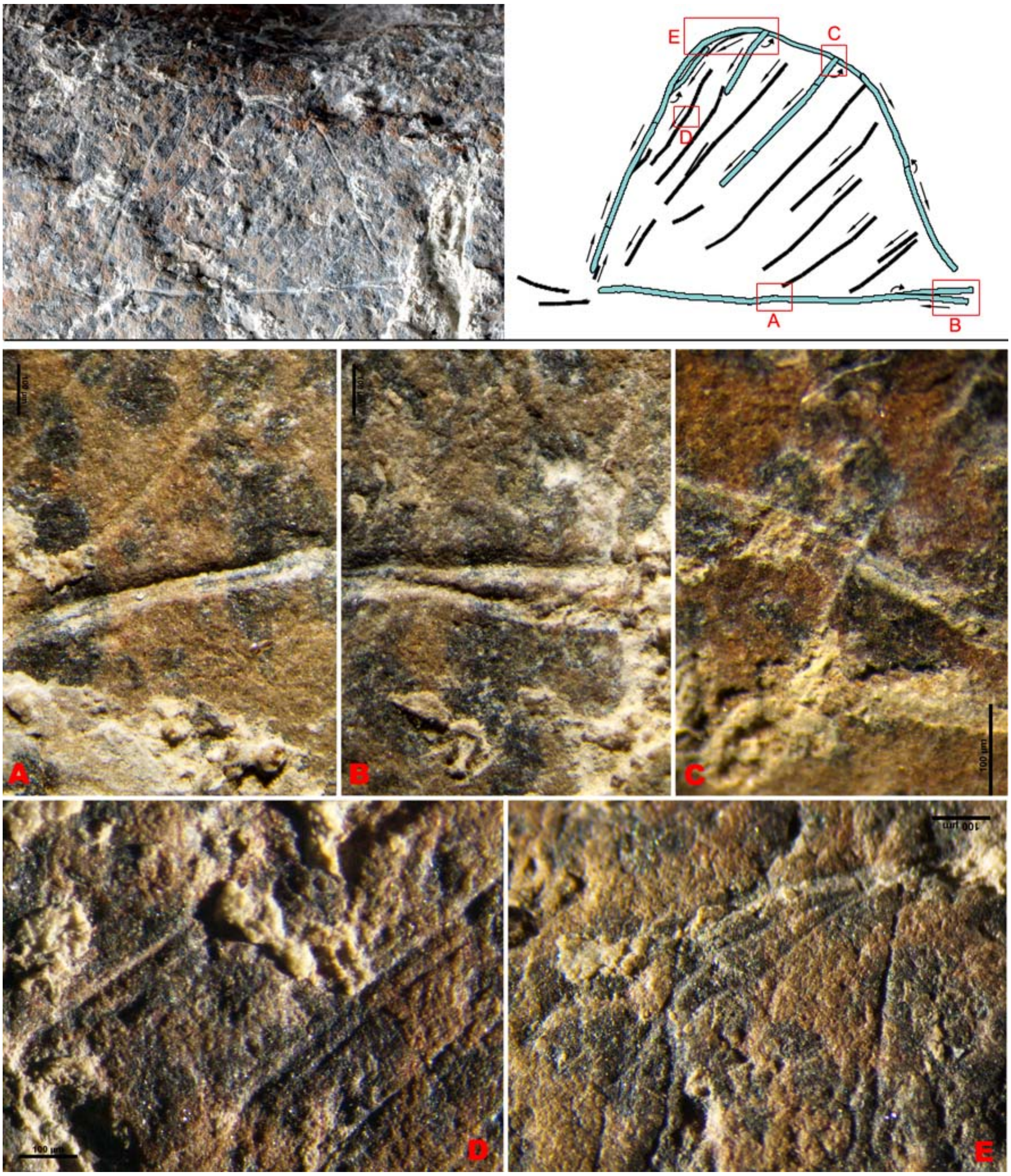

Fig F. Engraving process of the graphic unit E.

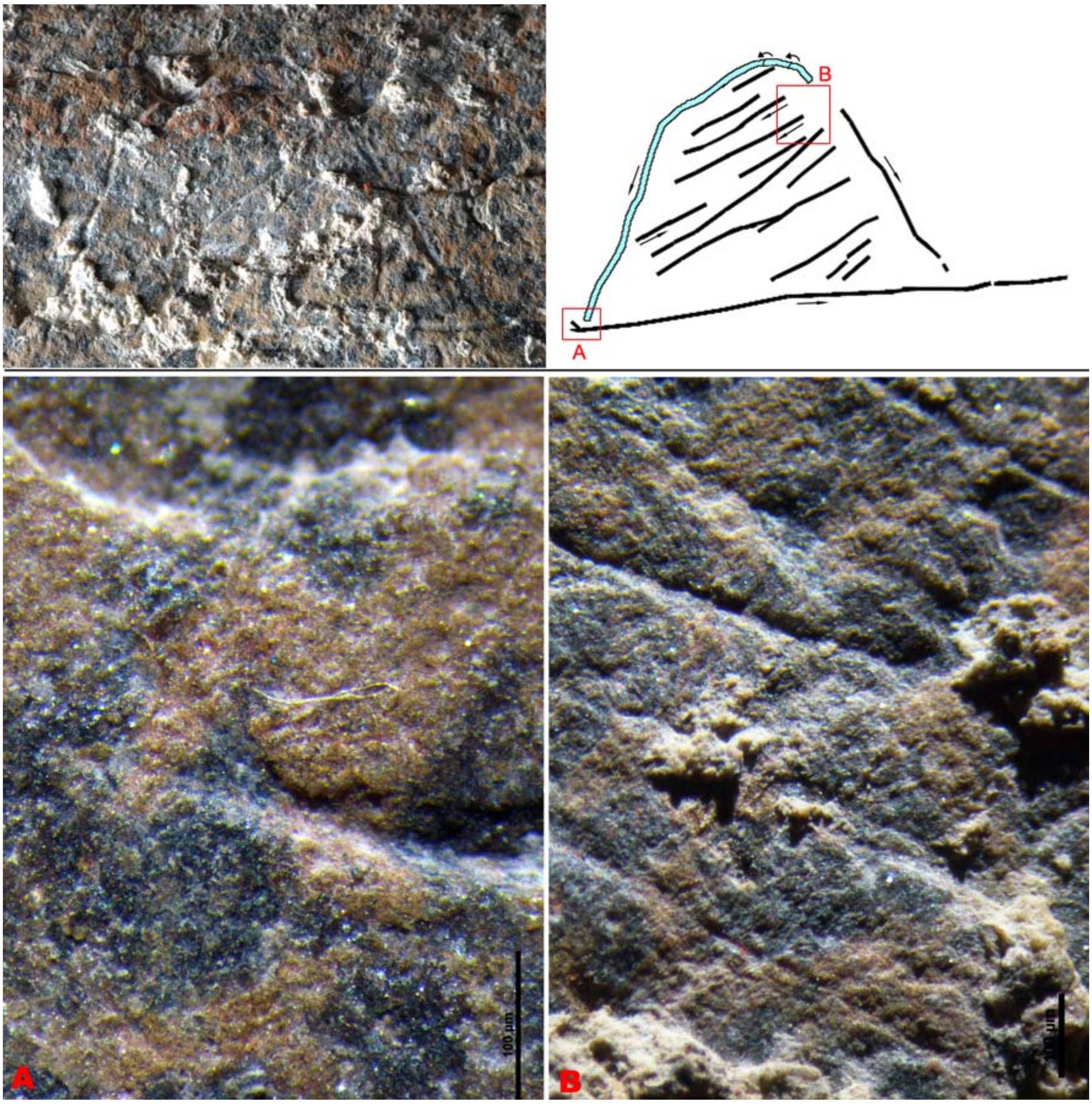

Fig G. Engraving process of the graphic unit F.

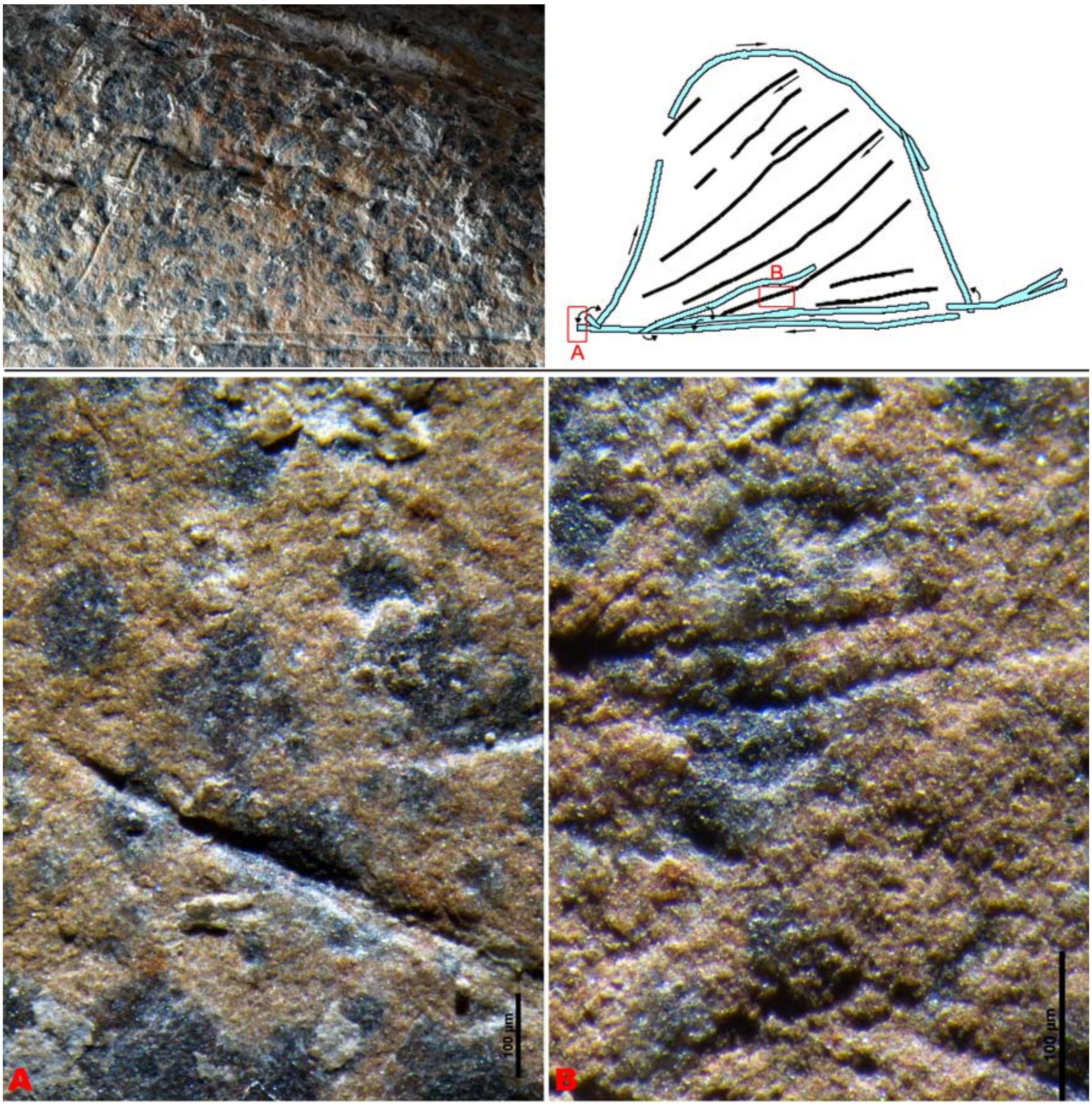

Fig H. Engraving process of the graphic unit G.
